# Structural Descriptors of gp120 V3 Loop for the Prediction of HIV-1 Coreceptor Usage

**DOI:** 10.1371/journal.pcbi.0030058

**Published:** 2007-03-30

**Authors:** Oliver Sander, Tobias Sing, Ingolf Sommer, Andrew J Low, Peter K Cheung, P. Richard Harrigan, Thomas Lengauer, Francisco S Domingues

**Affiliations:** 1 Max-Planck-Institute for Informatics, Saarbrücken, Germany; 2 British Columbia Centre for Excellence in HIV/AIDS, Vancouver, Canada; University of California San Diego, United States of America

## Abstract

HIV-1 cell entry commonly uses, in addition to CD4, one of the chemokine receptors CCR5 or CXCR4 as coreceptor. Knowledge of coreceptor usage is critical for monitoring disease progression as well as for supporting therapy with the novel drug class of coreceptor antagonists. Predictive methods for inferring coreceptor usage based on the third hypervariable (V3) loop region of the viral gene coding for the envelope protein gp120 can provide us with these monitoring facilities while avoiding expensive phenotypic tests. All simple heuristics (such as the *11/25 rule*) as well as statistical learning methods proposed to date predict coreceptor usage based on sequence features of the V3 loop exclusively. Here, we show, based on a recently resolved structure of gp120 with an untruncated V3 loop, that using structural information on the V3 loop in combination with sequence features of V3 variants improves prediction of coreceptor usage. In particular, we propose a distance-based descriptor of the spatial arrangement of physicochemical properties that increases discriminative performance. For a fixed specificity of 0.95, a sensitivity of 0.77 was achieved, improving further to 0.80 when combined with a sequence-based representation using amino acid indicators. This compares favorably with the sensitivities of 0.62 for the traditional *11/25 rule* and 0.73 for a prediction based on sequence information as input to a support vector machine and constitutes a statistically significant improvement. A detailed analysis and interpretation of structural features important for classification shows the relevance of several specific hydrogen-bond donor sites and aliphatic side chains to coreceptor specificity towards CCR5 or CXCR4. Furthermore, an analysis of side chain orientation of the specificity-determining residues suggests a major role of one side of the V3 loop in the selection of the coreceptor. The proposed method constitutes the first approach to an improved prediction of coreceptor usage based on an original integration of structural bioinformatics methods with statistical learning.

## Author Summary

HIV-1 cell entry requires a chemokine coreceptor in addition to the CD4 cell surface receptor. The two most common types of HIV coreceptors are called CCR5 and CXCR4. Whereas CCR5-using viral variants dominate directly after infection and during early stages of the disease, in about 50% of the patients, CXCR4-using variants appear in later stages of the disease, suggesting the coreceptor switch to be a determinant of disease progression. HIV coreceptors received substantial attention as antiviral drug targets, with CCR5 antagonists being currently tested in phase III clinical studies. Treatment with coreceptor antagonists requires continuous monitoring of coreceptor usage. The prominent role of coreceptors in disease progression and their potential as antiviral drug targets provides incentives for methodological improvements in coreceptor prediction and better understanding of the underlying determining factors regarding sequence and structural aspects. Our proposed method is the first approach to predict coreceptor usage based on structural information as opposed to established sequence-based methods. Including structural information improves predictive performance and is a first step towards a deeper understanding of the structural aspects of coreceptor usage.

## Introduction

### HIV Cell Entry and Coreceptor Usage

HIV virions enter human host cells through consecutive interaction with the CD4 cell surface receptor and one of the two major coreceptors CCR5 and CXCR4. After binding to CD4, a conformational switch in the surface protein gp120 of HIV reveals the coreceptor binding site, most notably the third hypervariable loop region V3. The V3 loop is considered to be the major viral determinant for coreceptor specificity [1]. After successful attachment to the host cell, fusion of the viral and host cell membranes takes place [2,3].

The coreceptor selectivity of the viral population is of central pathological and clinical importance.

Whereas in newly infected patients, CCR5-using (R5) variants dominate, in about 50% of the patients CXCR4-using (X4) variants appear during later stages of the disease characterized by progression towards AIDS. The cause of the observed coreceptor switch during progression is not fully understood; however, the close relation between the increase in the number of X4 variants and the decline of CD4_+_ cells and the disease progression towards AIDS is commonly agreed upon [4,5]. The categorization in R5 and X4 viral variants is highly correlated with but not identical to other categorization schemes into macrophage (M)-tropic and T cell line (T)-tropic or nonsyncytium-inducing versus syncytium-inducing variants [6].

### Monitoring Coreceptor Usage

Coreceptor antagonists are a new drug class, providing therapeutic options in addition to the established repertoire of protease and reverse transcriptase inhibitors [5,7]. Using a different mechanism and acting at a different stage of the viral life cycle, they provide new points of attack against multiresistant strains.

The observation that individuals carrying a 32-basepair (bp) deletion in the CCR5 coreceptor are highly resistant against HIV infection [8] specifically motivates the development of CCR5 antagonists.

Some CCR5 antagonists have proven safe and effective in phase II clinical trials [9] and are now being tested in phase III trials. While CCR5 inhibitors have already entered clinical testing, candidates for CXCR4 inhibitors are in earlier stages of development.

A major concern regarding drug treatment with CCR5 inhibitors is that it can select for the emergence of pre-existing or newly produced CXCR4-using variants [10,11]. The close relation with disease progression necessitates tight monitoring of coreceptor usage and possible switches while administering inhibitors for CCR5 or CXCR4.

Although phenotypic assays for monitoring coreceptor usage are commercially available, they are time-consuming and costly. To become a routine part of clinical diagnosis, inferring the phenotype from cheaper and faster genotypic analysis is desired. This approach has already entered routine clinical usage in resistance testing for the classical anti-HIV drug targets protease and reverse transcriptase [12].

Various methods for predicting phenotype based on sequence information are available. The most commonly used *11/25 rule* predicts a viral strain to be X4 in the presence of positively charged amino acids at positions 11 or 25 of the V3 loop [13]. More recently, methods based on statistical learning techniques have been developed, which show improved sensitivity in detecting X4 viral strains compared with the simple *11/25 rule* [14]. Neural nets [14], decision trees [15], support vector machines (SVMs) [15], and position-specific scoring matrices [1,16] have been applied, most of them significantly outperforming the simple *11/25 rule* [17].

### Structural Basis of Coreceptor Usage

**Figure 1:**
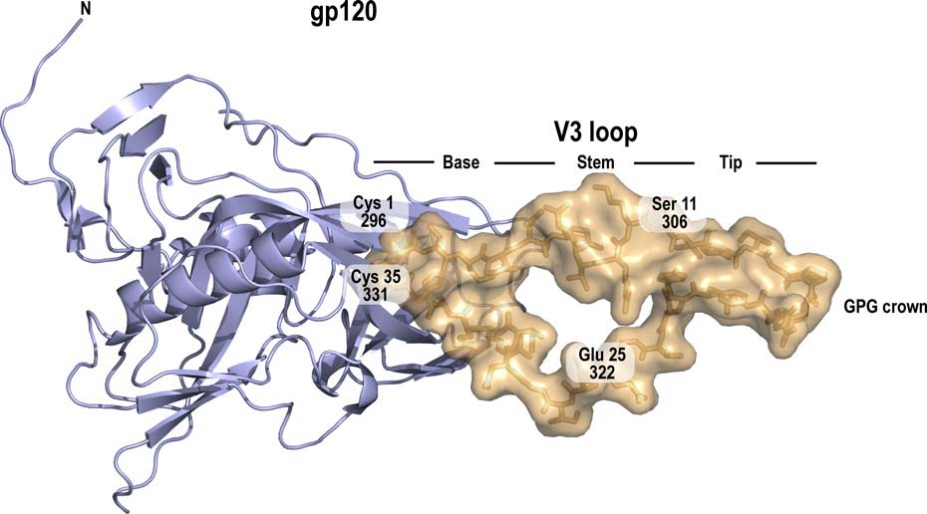
Crystal Structure of gp120 with the V3 Colored in Amber

To date, information on the three-dimensional structure of the V3 loop has not been exploited for predicting the coreceptor type used by a viral population. Including structural information can improve predictive performance and, even more importantly, be a first step towards a deeper understanding of the structural aspects of coreceptor usage. Several studies analyzed conformational properties of the V3 loop. However, these investigations did not particularly consider the impact on coreceptor usage. As Lusso [18] points out, structural understanding of coreceptor specificity is limited at the moment. In recent work, Watabe et al*.* [19] suggested empirical potentials to assess the fit of sequence variants to loop candidates generated by Monte Carlo variation of NMR peptide structures. So far, structural studies have been based on peptide structures, as no completely resolved structure of gp120 was available. The situation has changed with a recently published crystal structure of the HIV-1 JR-FL gp120 protein including the V3 loop by Huang et al. [20]. See Figure 1.

Although some evidence for conformational changes in the loop structure exists, there is an ongoing debate about the relevance of V3 loop conformation to coreceptor selectivity [21–23]. Sharon et al. [21] suggest that alternative conformations of the V3 loop play a key role in determining the coreceptor specificity of HIV-1. On the other hand, Scheib et al. [22] argue that there is a predominant conformation for both R5 and X4 variants and that varying sequence features are responsible for specificity towards the respective coreceptor.

### Novel Structural Descriptor and Related Methods

**Figure 2:**
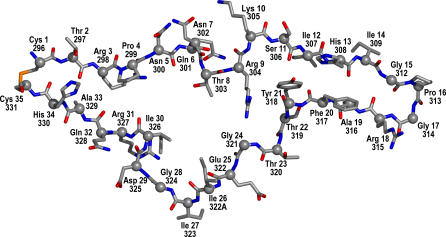
V3 of gp120 with Residue Labels Labels in the first row are amino acid identifiers and V3 sequence reference numbers (relative to the subtype B consensus sequence of length 35), labels in the second row are PDB file residue numbers in chain G of 2b4c. The loop has the same orientation as in Figure 1.

Here, we describe the first structure-based approach to predicting HIV-1 coreceptor usage. In particular, we propose and evaluate a novel structural descriptor for capturing the spatial distribution of five functionally defined atom types in the V3 loop (see Figure 2).

In a practical scenario, only sequence data but no structures will be available for different viral variants. Thus, we chose to evaluate two approaches: (1) to use a simple descriptor (*V3SD_Cβ_*), which approximates the position of all functional side chain atoms by the fixed Cβ positions of the structure 2b4c [20]; and (2) a descriptor *V3SD_scwrl_,* which uses the crystal structure 2b4c [20] as a rigid backbone template for the V3 loop region and models side chains using SCWRL [24]. SCWRL is a reliable and fast program to predict side chains for large sets of sequences. By comparing the descriptors *V3SD_Cβ_* and *V3SD_scwrl_,* which represent structures of viral variants at two different levels of approximation, the tradeoff between increased uncertainty and the improved information about side chain location and length can be assessed.

To specifically address the structural uncertainty in the presence of insertions and deletions, we evaluate the performance separately for sequence variants with substitutions only, as opposed to variants also exhibiting insertions and deletions relative to the reference V3 loop of the structure 2b4c. To derive structural descriptors from the modelled variants, the side chains are represented by functional atoms, labelled as hydrogen-bond donor, acceptor, ambivalent donor/acceptor, aliphatic, or aromatic ring, according to Schmitt et al. [25]. For the subsequent prediction based on an SVM, the spatial arrangements are encoded by 15 distance distributions, one for each pair of functional atom types. Thus, for each atom-type combination (e.g., donor–donor, donor–acceptor, . . .) all Euclidean distances between the respective atoms are computed and condensed into a distribution function, similar to a smoothed histogram.

**Figure 3:**
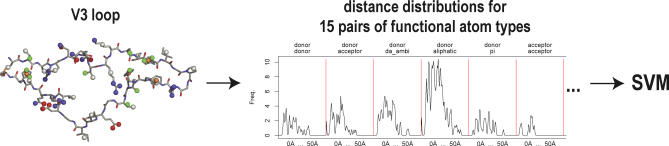
Schematic Overview of the Structural Descriptor Computation and Coreceptor Usage Prediction

The set of 15 distance distributions is used as vectorial input to the SVM. See Figure 3 for a schematic overview and the section Structural Descriptors for methodological details.

The proposed structure representation is related to ideas from protein structure comparison and prediction. Distributions of atomic distances have been used successfully in structure comparison [26,27]. In protein structure prediction, distributions of distances have been applied as knowledge-based potentials to evaluate the fit of a sequence to a specific structure [28,29].

In the context of protein function, Stahl et al. [30] have used distance-based descriptions to cluster active sites of enzymes based on chemical and geometric properties. For the analysis of protein–protein interaction interfaces, Mintseris and Weng [31] have proposed atomic contact vectors which consist of contact counts derived from thresholded distance matrices. Aloy and Russell [32] have suggested empirical potentials to assess the compatibility of a pair of sequences to the contacts formed in a known complex of two respectively homologous sequences. In a similar setting, MULTIPROSPECTOR [33] uses a threading algorithm to align a pair of sequences to a structurally resolved protein–protein complex. In addition to the interface energy term as in [32], this method also uses the threading score for the protomers themselves.

Structural understanding in the present problem is seriously hampered by the fact that structural details on complexation with the coreceptor are unknown. This is why we refrain from an attempt to integrate structural information on the coreceptors. Another aggravating factor is that no crystal structures are available for viral variants. As it is unlikely that comprehensive structural data on the wealth of viral variants will become available, modelling of side chains, and potentially also changes in the backbone, is necessary.

## Results/Discussion

### Predictive Performance of Sequence-Based and Structural Descriptors

To assess the predictive performance of the structure-based descriptors, we compared the two variants *V3SD_Cβ_* and *V3SD_scwrl_* against purely sequence-based predictions by the *11/25 rule,* which predicts X4 in the presence of positively charged residues at positions 11 or 25, and *Indicator*. *Indicator* performs prediction based on an SVM using a binary sequence encoding, which uses a bit-vector to indicate the presence or absence of a specific amino acid at a specific V3 loop sequence position. We evaluated the two structural descriptors and the two sequence-based predictors on data compiled from the Los Alamos HIV Sequence Database and several publications [14,34–37]. The evaluation is performed on a dataset containing 514 mutually distinct V3 sequences (SEQ_indels,514_) and a smaller subset, containing 432 sequences without indels (SEQ_noindels,432_). Each of the sequences is annotated as either using CCR5 only or being capable of using CXCR4. See Materials and Methods for methodological details and the Dataset and sequence alignment section for a description of the dataset.

For measures of performance we used the sensitivity at the specificity of the *11/25 rule,* the area under the ROC curve (AUC), the accuracy at a cutoff of 0.5 (for the posterior probability obtained by the SVM), and the positive predictive value (PPV) at the specificity of the *11/25 rule*. Of all these measures, we consider the sensitivity at the specificity of the *11/25 rule* as most important in practice, because it focuses on detecting X4 viral variants at an acceptable level of false positives (R5 erroneously considered to be X4). See the section Evaluation and definition of performance measures for definitions of the performance measures.

**Figure 4:**
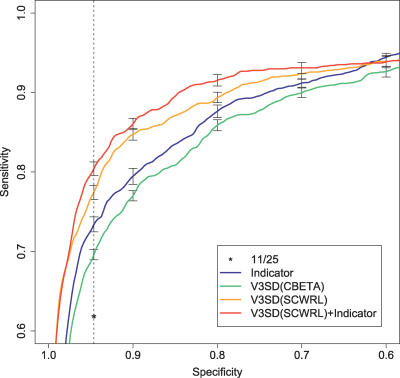
ROC Comparison of Predictive Performance: Sequence-Based Predictions (*11/25 rule* and *Indicator*) and Structural Descriptors (*V3SD*_Cβ _and *V3SD_scwrl_*) on the Dataset *SEQ_noindels,432_*

Figure 4 contains ROC (receiver operating characteristic) curves for a performance comparison of the methods. ROC curves plot (1-Specificity) against Sensitivity for varied decision cutoffs, ranging from predicting mainly R5 (towards the lower left corner) to predicting mainly X4 (towards the upper right corner). On our dataset (see the section Dataset and sequence alignment for details), the *11/25 rule* has a sensitivity of 0.6186 while exhibiting a specificity of 0.9463. Considering the routine clinical application of this simple rule, the benefit of improving the sensitivity towards X4 viral variants is obvious. For the fixed specificity of 0.9463 (i.e., maintaining a fixed number of false positives), the sequence-based indicator prediction using a linear SVM improves sensitivity to 0.7340. A similar improvement has been reported previously [15,17] when applying statistical learning methods in comparison to the traditional *11/25 rule*.

For the simpler form of structural descriptor *V3SD_Cβ_ ,* the performance is below the *Indicator* prediction at a sensitivity of 0.6959. Still, this constitutes a considerable improvement over the *11/25 rule*. Thus, as features different from pure sequence information are encoded in this structural descriptor, its analysis can provide important insights regarding structural features.

Using structural models for the sequence variants with side chains placed by SCWRL [24], predictive performance improves considerably over the simple structural descriptor *V3SD_Cβ_* and even compared with the *Indicator* encoding. The structural descriptor *V3SD_scwrl_* improves sensitivity to 0.7742. SCWRL faces a hard task in optimizing side chain conformations as no direct contacts between the side chains within the loop with side chains of binding partners are present. However, the improved predictive performance indicates that the additional information over the *V3SD_Cβ_* descriptor helps in discriminating coreceptor usage.

One important aspect might be the information about side chain length and volume, which is completely lost in the *V3SD_Cβ_* descriptor.

An overview of predictive performance for further measures can be found in Table 1. The observed ordering of methods regarding performance are similar to the trend observed for the sensitivities. The absolute performance increases regarding AUC and accuracy are smaller. This is because AUC and accuracy are less responsive to improvements in detection of X4 variants due to the class imbalance towards R5 samples.

**Table 1:**
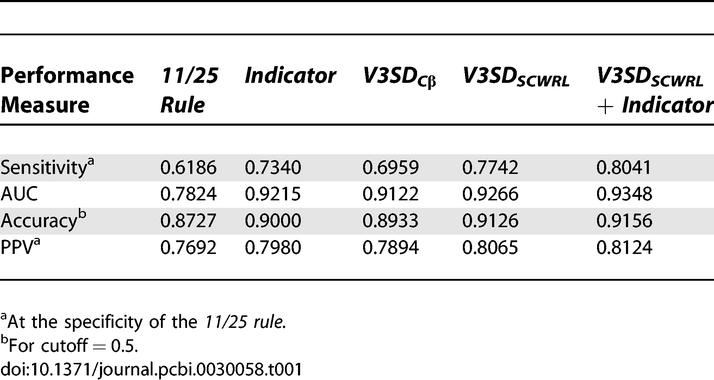
Performance on the Dataset SEQnoindels,432

In Table 2 the statistical significance of relative sensitivity improvements between methods is tabulated. The improvement from the *11/25 rule* to the *Indicator* is significant at a *p*-value of 0.0059 (paired Wilcoxon test), as is the improvement of *V3SD_scwrl_* over *Indicator* (0.0137). The error bars in Figure 4 are nonoverlapping for the sensitivities at the specificity of the *11/25 rule* (dashed line). This also indicates significant differences in predictive performances.

**Table 2:**
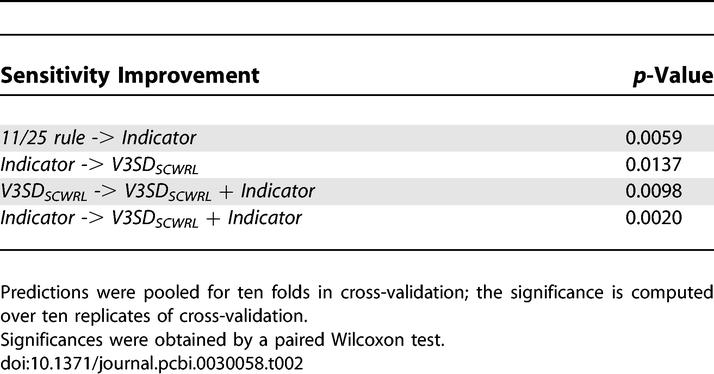
Significance of Sensitivity Improvements at the Specificity of the *11/25 Rule*

### Combining Structural Descriptors with Sequence-Based Representations

Considering the different type of information in the sequence-based and the structural descriptors, we combined the respective features to assess whether further predictive improvements are feasible. The sequence-based and structural features were combined by concatenating the corresponding feature vectors. As seen in Figure 4, combination of the sequence-based *Indicator* encoding and the structural descriptor *V3SD_scwrl_* further improves sensitivity to 0.8041 at the specificity of the *11/25 rule* (0.9463). This indicates that sequence and structure convey complementary information, to some extent. See Table 1 for further performance measures and Table 2 for a significance assessment of the relative improvement.

For a fixed specificity of 0.9, a similar increase from sequence-only to structure-based descriptors can be observed: 0.7946 (*Indicator*), 0.8474 (*V3SD_scwrl_* ), 0.8603 (*V3SD_scwrl_* + *Indicator*).

### Viral Variants with Indels Relative to 2b4c

**Figure 5:**
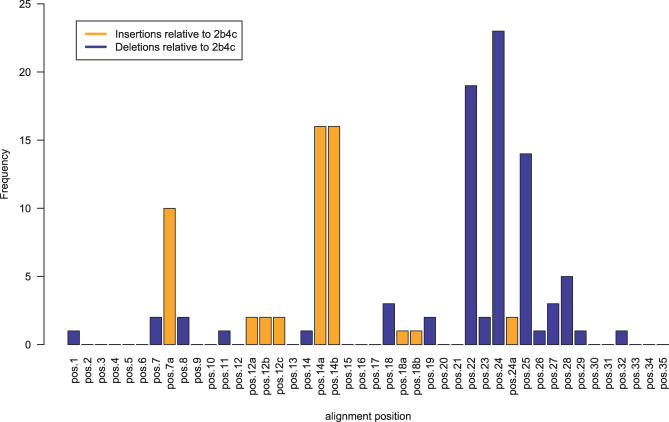
Distribution of Insertions and Deletions over the V3 Loop The *x*-axis labels denote V3 sequence reference numbers (relative to the subtype B consensus sequence of length 35) like those annotated in Figure 2. Positions 7a, 12a, 12b, 12c, 14a, 14b, 18a, 18b, and 24a are insertions relative to the structure PDB 2b4c as well as the subtype B consensus sequence.

The previous performance assessment was done only on viral variants without insertions or deletions relative to the V3 region of 2b4c. However, for broad applicability it is desired to cover sequences with indels as well. Investigating the positions of observed insertions and deletions shows that they are not uniformly distributed along the V3 region. Instead, there are preferences for certain positions. Figure 5 illustrates the positional distribution of insertions and deletions.

Around position 7, insertions and a few deletions can be observed. After position 12 there is a rare three-residue deletion, occurring in two sequences in our dataset. Between positions 14 and 15 there is a rather common two-residue insertion. The effect of this insertion on the β pairing within the hairpin is unclear; it might disrupt the pairing. A rather common deletion is observed at position 22. Higher rates of insertions and deletions can be found around position 24, the bulgy middle region. In this neighborhood it appears to be easier to structurally adapt to insertions and deletions by slight conformational changes.

For sequence variants containing insertions relative to the V3 region of 2b4c, the inserted residues were ignored in the descriptor. For variants with deletions, only the remaining residues contributed to the descriptor.

For insertions as well as deletions, no remodelling of the backbone or loop closure was performed.

We compare the sensitivity at the specificity of the *11/25 rule* for the full dataset including indels with the performance reported above in the section Predictive Performance of Sequence-Based and Structural Descriptors. Whereas the sensitivity of the *11/25 rule* drops to 0.5782, the performances for *Indicator* (0.7182), *V3SD_scwrl_* (0.7712), and for the combination of *Indicator* and *V3SD_scwrl_* (0.8052) change only slightly. This shows that the proposed structural descriptor is sufficiently robust to handle sequence variants containing indels. See Protocol S1 for additional material on viral variants with indels.

### Identification of Discriminating Structural Features

To assess the importance of features in the structural descriptors, we used three approaches for scoring how characteristic the respective features are for each coreceptor class. First, we analyzed the separation of the two coreceptor classes by each feature using the Wilcoxon test-statistic (*Wilcoxon*). Second, the ratios of feature variability between and within the two coreceptor classes were assessed (*variation ratio*). Third, a random forest classifier was used to estimate the feature importance of each feature (*RF importance*).

**Figure 6:**
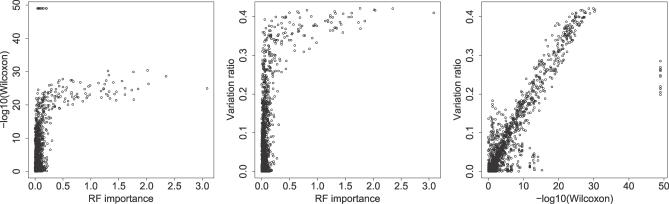
Pairwise Scatter Plots of the Feature Importance Measures *Wilcoxon*, *Variation Ratio*, and *RF Importance* against Each Other

**Figure 7:**
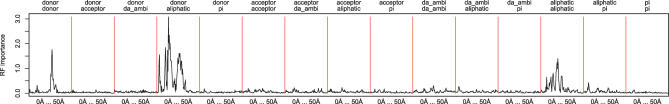
Random Forest Feature Importance Measured by the Mean Decrease in Gini Coefficient

Random forests are predictive classifiers and were applied as substitutes for the SVMs above, because their construction as an ensemble of decision trees allows the extraction of feature importance measures. See Figure 6 for pairwise scatter plots of the three importance measures and Figure 7 for an illustration of *RF importance*. Finally, we investigate the relevant residue pairs contributing to the characteristic features.

#### Wilcoxon separation of coreceptor types.

The *Wilcoxon* score highlights donor–aliphatic distances and aliphatic–aliphatic distances similar to the random forest evaluation. For donor–aliphatic distances, the important intervals are 2.5–4, 9–10, 12.5–18, and 25.5–26 Å. For the aliphatic–aliphatic distances 10–15, 19–21 Å are the most outstanding intervals, while acceptor–acceptor distances at several distinct peaks appear at 5.5–7.5, 15–16, 24, and 34 Å. See Protocol S1 for feature importance according to the *Wilcoxon* score.

#### Ratio of feature variation between and within groups.

The *variation ratio* score shows a high correlation with the *Wilcoxon* score. The top 50 features are donor–aliphatic distances in the intervals 2.5–4, 8.5–10, 12.8–17.5, and 23.5–28.5 Å, as well as aliphatic–aliphatic distances 7.5–8, 10.5–15, 19.5–21, and 24.5–26 Å. See Protocol S1 for feature importance according to the *variation ratio* score.

#### Random forest feature importance.

On our dataset, random forests yield predictions with a performance close to the performance of the nonlinear SVM used above in the section Predictive Performance of Sequence-Based and Structural Descriptors. However, random forests facilitate feature interpretation by scoring features with an importance measure (mean decrease in Gini coefficient). Compared with the two feature assessment scores above, *RF importance* provides a multivariate evaluation, considering also mutual relationships between features with respect to the predictive model.

In the *RF importance* analysis, three feature groups stand out (see Figure 7). The 50 highest-scoring features regarding mean decrease in Gini coefficient are all from the three groups donor–aliphatic, aliphatic–aliphatic, and donor–donor. Donor–aliphatic distances provide important features over a broad range of distances. Most outstanding distance intervals are: 4, 9–18, 23–30, and 33 Å. Aliphatic–aliphatic are similarly important over a wide distance range: 7, 12–14, 19–21, and 25 Å. The importance of donor–donor distances shows a distinct peak at around 28 Å. In contrast to the *Wilcoxon* importance ranking, acceptor–acceptor distances are not considered to be highly important.

Correlating the *Wilcoxon* scores with the *RF importance* scores reveals that all of the top 50 RF features have a *Wilcoxon* score above 20 (see Figure 6). This indicates that a high *Wilcoxon* separation is required for a high *RF importance* score, but not vice versa. In general, the score variabilities for the *Wilcoxon* and *variation ratio* scores are higher than for the *RF importance* score; however, the top 50 features are similar in all three scoring schemes.

#### Identification of residues contributing to important features.

For each of the important distance intervals highlighted above and for each pair of residue types, we examine which residue pairs of the given type contribute to respective distance intervals. The analysis is performed for the four donor–aliphatic distance intervals 4 ± 0.5, 9 to 18, 23 to 30, and 33 ± 0.5 Å; the four aliphatic–aliphatic distance intervals 7 ± 0.5, 12 to 14, 19 to 21, and 25 ± 0.5 Å; and donor–donor atoms in the distance of 28 ± 0.5 Å. For each of these intervals we compute a measure of relevance for residue pairs.

**Figure 8:**
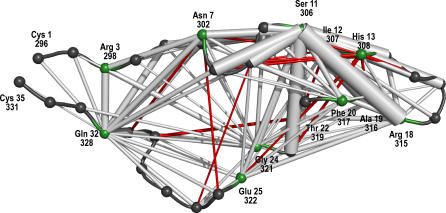
Importance of Residue Pairs for Donor–Aliphatic Pseudo-Atoms at Distances between 9 Å to 18 Å The width of the edges is proportional to the difference in contributions to the descriptor features of the two coreceptor classes. Edges in gray denote residue pairs characteristic for X4 variants, edges in red mark residue pairs contributing to R5-specific descriptors. Residues that are considered to be important for several distance intervals are marked in green. The loop has the same orientation as in Figures 1 and 2.

The measure consists of the fraction of X4 variants in which this pair contributes to the respective interval minus the fraction of R5 variants in which this residue pair contributes. As shown in Figure 7, donor–aliphatic pseudo-atoms at distances between 9 to 18 Å are most prominent regarding *RF importance* score. See Figure 8 for a graphical representation of relevant residues and residue pairs at this distance interval. Edges between two residues are scaled by the contributions of this residue pair to the respective distance range. Edges colored in gray are pointing to X4 variants, whereas edges colored in red characterize residue pairs specific for R5 strains. Residue 11(306) contributes to several characteristic features in various residue pairings. The dominant impact of residue 11(306) reflects its role in the *11/25 rule* and agrees with other univariate residue importance studies [17]. Further relevant residues are 3(298), 7(302), 13(308), 18(315), 20(317), 22(319), 24(321), 25(322), and 32(328). Interestingly, residues 22(319) and 13(308) seem to be overrepresented for R5 viral variants, whereas the other residues are mainly indicators of X4 strains. See Protocol S1 for further tabulation of relevant residue pairs.

The other distance intervals mainly confirm the relevant residues found above. In addition, for aliphatic–aliphatic pseudo-atoms at distances between 7 Å ± 0.5 Å, a close coupling of R5-specific features for residues 12(307) and 19(316) are highlighted. Residue 27(323) exhibits several weak R5-indicating pairings for aliphatic–aliphatic pseudo-atoms at distances between 25 Å ± 0.5 Å as well as for donor–aliphatic atoms at distances between 23 Å to 30 Å. Donor–donor distances confirm the donor–aliphatic results, similar key residues are clearly highlighted here.

Except for residue 19(316), the side chains of all relevant residues in the β hairpin tip are on the same side of the loop (pointing outwards from the paper plane in Figure 2), suggesting a major direct or indirect role of this side (called upside) of the tip in determining the selectivity towards the two coreceptor types. At residue 19(316), R5 variants have more hydrogen donor or acceptor groups compared with X4 variants. X4 variants are more aliphatic and have fewer acceptors at position 18(315) compared with R5 variants. For residue 20(317), X4 variants have less pi and more donor groups relative to R5. For the remaining residues of the loop, we observe a slight tendency towards aliphatic residues pointing to the upside of the loop relative to the hairpin tip, in particular in residues 7(302), 11(306), 24(321), and 32(328). Interestingly, most of the features highlighted in the analysis above are indicators for X4 variants, only a few are descriptive of R5 strains.

### Conclusion and Outlook

The proposed descriptor yields a considerable performance increase over the established *11/25 rule* and even compares favorably with newer methods based on statistical learning (*Indicator*). In contrast to purely sequence-based coreceptor usage predictions, the proposed structural representation captures the relative three-dimensional arrangement of chemical groups. From a biophysical perspective, this relative placement of chemical groups is determining which coreceptor the viral variant will bind. Due to its robustness with respect to sequence variants containing indels, it can be applied in realistic scenarios and on large-scale datasets. The most interesting aspect of the proposed descriptor is its integration of structural data, providing the first application of structural data in the context of coreceptor usage prediction. The combination of methods from structural bioinformatics with statistical learning methods allows for competitive performance as well as interpretation of coreceptor usage at the structural level.

Despite its good performance, there are several limitations and possible directions for improvement, either by methodological enhancements or by integration of further experimental data. As almost no side chain interactions take place within the V3 loop and the binding partner is not available in the structural model, SCWRL faces a difficult task in optimizing side chains. One possible way of relaxing this difficulty is by considering ensembles of alternative side chain conformations in the structural descriptor. From a methodological point of view, alternative conformations are easy to integrate into the distance distributions in a weighted manner. A further possible bottleneck is the assumption of a fixed backbone structure. Further understanding of the structure–function relationship of coreceptor usage or new insights in the debate mentioned above [21–23] could be incorporated into the descriptor. Instead of the fixed backbone structure, several alternatives are possible. Experimentally resolved peptide structures could be used to model sequence variants or molecular dynamics simulations could be used to generate ensembles of backbone variants. With all these alternatives, the proposed descriptor provides a generic way of incorporating new structural information on V3 loop conformation; especially interesting would be crystal structures of X4 viral variants.

Another interesting perspective is to correlate the discriminative spatial features of the V3 region to spatial arrangements in the coreceptor. Published chemokine receptor models [38,39] could be used to generate such spatial descriptions and to search for complementary arrangements of physicochemical properties. Finally, the proposed method to describe the spatial arrangement of physicochemical properties is not limited to the demonstrated application, in principle. By providing a vectorial representation of a binding site, it can be used as a generic way of describing and comparing any set of binding sites regarding geometric and physicochemical features involved in different protein–protein interactions.

## Materials and Methods

### Dataset and sequence alignment.

From the HIV Sequence Database at Los Alamos National Laboratory and several publications [14,34–37], we obtained 1,100 clonal samples with annotated coreceptor phenotype from 332 patients. To reduce the risk of positively biased results, we removed all duplicate V3 sequences (i.e., sequences with 100% sequence identity to another sequence in the dataset), resulting in 514 mutually distinct sequences. For each of the samples, the coreceptor phenotype is denoted as R5, X4, or R5/X4. R5/X4 are viral strains being capable of using either of the two coreceptors. R5/X4 and X4 variants were pooled into a single class (called X4 in the sense of X4-capable), as opposed to variants that are limited to using CCR5 (called R5 in the sense of R5-only). The dataset after duplicate removal contains 363 R5 and 151 X4 samples.

We aligned these sequences using the multiple alignment package MUSCLE [40] with default parameters. Visual inspection showed no obvious degeneracies or problems in the alignment. The alignment of this sequence dataset (called SEQ_indels,514_) shows that 82 sequences contain insertions and deletions relative to 2b4c. By restricting the set SEQ_indels,514_ to V3 variants without indels relative to the V3 region of 2b4c, we obtained 432 mutually distinct V3 loop sequences (called SEQ_noindels,432_). Of those sequences, 97 are X4 variants, 335 are R5 strains.

### 11/25 charge rule and indicator sequence encoding.

The traditional *11/25 rule* is an empirically derived procedure routinely used in clinical practice to predict coreceptor usage. It predicts a viral variant to be X4 if there is a positively charged amino acid at V3 position 11 or 25 [13]. Among simple sequence rules (i.e., not based on statistical learning), Resch et al. consider the *11/25 rule* to be the best predictor of coreceptor usage [14].

Various statistical learning methods were used to improve predictive performance [1,14,15]. Here we use linear SVM prediction based on an indicator encoding of the sequences (*Indicator*) [17]. A viral variant is encoded by an indicator vector (consisting of only zeros and ones). Each component in this vector indicates the presence or absence of a specific amino acid at a specific V3 position.

### Structural descriptors.

The protein structure of the HIV-1 JR-FL gp120 protein including the V3 loop (Protein Data Bank (PDB) structure 2b4c [20], based on a CCR5-using JR-FL variant) was retrieved from the RCSB PDB (http://www.pdb.org). The V3 loop in chain G ranging from residues 296 to 331 was extracted. Based on this loop backbone, we model the side chain positions for each sequence variant using SCWRL [24]. As no structure information for the sequence variants is directly available, we chose to evaluate two approaches: (1) to use a simple descriptor (*V3SD_Cβ_*), which approximates the position of all functional side chain atoms by the fixed Cβ positions of 2b4c; and (2) a descriptor *V3SD_scwrl_,* which is based on modelled side chains. This way the tradeoff between increased uncertainty and the improved information about side chain location and length can be assessed.

We then represent the side chains by five functional atom types, labelled as hydrogen-bond donor, acceptor, ambivalent donor/acceptor, aliphatic, or aromatic ring. Amino acids R, N, Q, K, and W are classified as donors. Acceptors are N, D, Q, and E. Ambivalent donor/acceptors comprise H, S, T, and Y. As aliphatic amino acids, we consider A, R, C, I, L, K, M, P, T, and V. Pi-stacking centers are H, F,W, and Y. This definition follows [25], but does not assign backbone centers as pi-stacking. For aliphatic and aromatic interaction centers, all involved atom positions were averaged per residue to compute a pseudo-atom. In contrast to [25], who weight atoms by their solvent access for computing the pseudo-atom of aliphatic side chains, we used the unweighted average of the respective carbons as the solvent exposure of the V3 loop, which can be seen as rather uniform.

For the subsequent statistical analysis, the spatial arrangement of these functional properties is encoded by distance distributions. For each of the 15 combinations of functional atom types (i.e., donor–donor, donor–acceptor, etc.), pairwise Euclidean distances between the respective pseudo-atoms in the V3 loop are calculated. Note that the number of these distances depends on the number of pseudo-atoms in the two groups. From these distance matrices, we derive distance distributions using a kernel density estimate with Gaussian kernel and bandwidth of 1 Å. The density estimates are then discretized by uniform sampling at intervals of 0.5 Å, resulting in a 15 (distance distributions for atom type combinations) times 100 (sample points) dimensional vector. The resulting vector is used as a structural descriptor for a given sample, as an alternative to the purely sequence-based indicator representation, and used as input to the statistical learning method. The bandwidth as well as the sampling intervals for the distance-based descriptors have been set to reasonable values based on empirical observations. To keep computation times feasible, they were not optimized systematically.

### Prediction based on support vector machines.

For the sequence indicator encoding (*Indicator*), a linear kernel is used, as previous studies showed that nonlinear kernels do not help for simple sequence encodings [17]. For prediction based on the structural descriptors, a radial basis function kernel [41] is applied, as it provides better performance than a linear kernel in this case. In both cases probabilistic predictions are obtained from the SVM by the method of Platt [42] to get estimates of prediction confidence and a scoring classifier for the ROC analysis.

To optimize SVM parameters, we conducted parameter grid searches. For the linear kernel (*Indicator*), we varied the cost parameter log_2_ C in [-7, 2]. For the radial kernel (*V3SD_Cβ_, V3SD_scwrl_*), we varied the cost parameter log_2_ C in [-6, 5] and the gamma parameter log_2_ γ in [-15, −5]. Optimal parameter values were obtained from ten bootstrap samples of the dataset and kept fixed for the subsequent analysis and evaluation. Each bootstrap sample contained 9/10 of the number of samples in the original dataset (drawn with replacement), using the default in the R package e1071 [43].

### Evaluation and definition of performance measures.

To assess predictive performance for the structural descriptors, we performed ten replicates of 10-fold cross-validation. Evaluation of predictive performance was done using ROCR [44]. The measures used for evaluation of predictive performance are sensitivity at the specificity of the *11/25 rule*, AUC, accuracy, and PPV. In the following, Ŷ denotes the predicted coreceptor class and Y is the coreceptor actually used for a sample. P and N denote the number of positives (X4) and negatives (R5). TP and TN denote the number of correctly predicted positives and negatives, and FP and FN denote the number of samples incorrectly predicted as positive or negative, respectively. Sensitivity is defined as P(Ŷ = X4|Y = X4) and is estimated as TP/P. The specificity P(Ŷ = R5|Y = R5) is estimated as TN/N. The AUC is calculated by adding the area of trapezoid strips under the ROC curve. This is equal to the value of the Wilcoxon-Mann-Whitney test statistic and also to the probability that the classifier will score a randomly drawn positive sample higher than a randomly drawn negative sample [45]. The accuracy is defined as P(Ŷ = Y) and estimated by (TP + TN)/(P + N) at the cutoff 0.5 for the posterior class probability. The PPV P(Y = X4| Ŷ = X4) is estimated as TP/(TP + FP).

### Measures for feature interpretation.

For evaluation of the importance of the features in the structure-based descriptor, we used three scoring schemes. First we used the -log_10_(*p*-value) of the Wilcoxon rank-sum statistic (*Wilcoxon*) [46]. As a second measure we utilized the ratio of feature variation between and within groups (*variation ratio*), which is frequently used in gene ranking for microarray analysis [47]. For a feature *i,* this ratio is

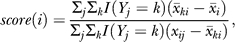
where 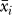 and 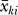 denote the average of feature *i* across all samples and across samples belonging to class *k* only.

Third, we used the random forest feature importance scores (*RF importance*) based on the mean decrease of the Gini index [48].

### Software and computational details.

 Computations on the sequence variants and structural models were performed using Python and Biopython [49]. For computationally intensive parts, Grid Engine was employed for running the analysis on a compute cluster. Analysis of the results and prediction was performed using the statistical language R [50] with the packages e1071 [43], randomForest [51], and ROCR [44]. Protein structure visualizations in Figures 1, 2, 3, and 8 were created using PyMOL [52]. The source code for prediction and analysis is available upon request.

## Supporting Information

Protocol S1 Supporting Figures and Tables (6.6 MB PDF)

### Accession Numbers

The PDB (http://www.pdb.org) accession number used in this paper is for gp120 (2b4c, chain G).

## Abbreviations

AUC: area under the ROC curve
M: macrophage
PPV: positive predictive value
ROC: receiver operating characteristic
SVM: support vector machine
T: T cell line
V3: third hypervariable
X4: CXCR4-using
