# Supplementary material for: Structural Descriptors of gp120 V3 Loop for the Prediction of HIV-1 Coreceptor Usage
Source: PLoS Comput Biol. 2007 Mar 30;3(3):e58. doi: 10.1371/journal.pcbi.0030058 (PMC1848001; doi:10.1371/journal.pcbi.0030058)
Supplement: Protocol S1 — (6.6 MB PDF) [file pcbi.0030058.sd001.pdf]

Supporting Information for
Structural Descriptors of gp120 V3 Loop for the Prediction of HIV-1 Coreceptor Usage

Oliver Sander[1], Tobias Sing[1], Ingolf Sommer[1], Andrew J. Low[2],
Peter K. Cheung[2], P. Richard Harrigan[2], Thomas Lengauer[1], Francisco S. Domingues[1]

[1]Max-Planck-Institute for Informatics, Saarbrücken, Germany

[2]British Columbia Centre for Excellence in HIV/AIDS, Vancouver, Canada

January 04, 2007

1 V3 loop structure in 2b4c

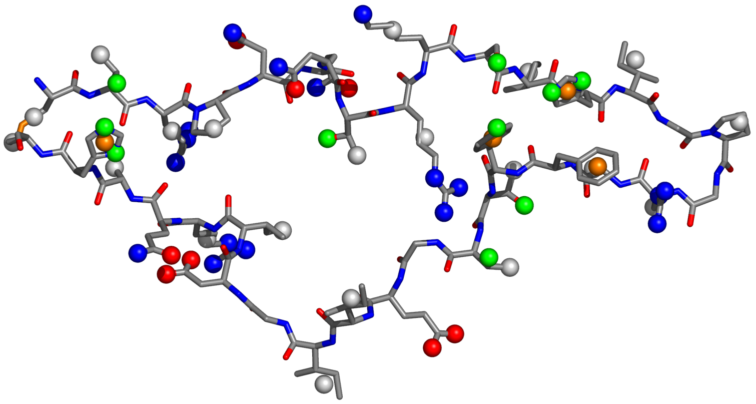

Figure 1: V3 loop structure of 2b4c with functional pseudo-atoms marked in blue (donor), red (acceptor), green (ambivalent donor/acceptor), white (aliphatic), and orange (pi).

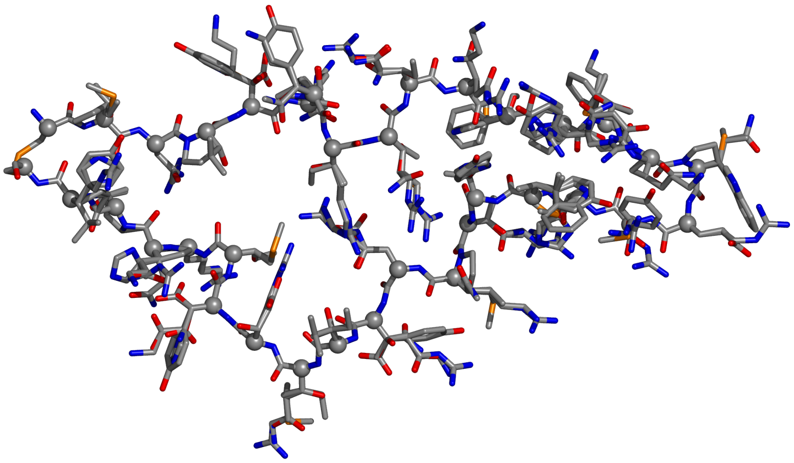

Figure 2: Superimposition of modelled V3 loop structures of all 432 viral variants without indels relative to PDB 2b4c.

2 Data set summary

Table 1: Tabular summary of data set characteristics.

data set		
$SEQ_{noindels,432}$	number of sequences	432
	sequence length (const.)	35
	number of patients	257
	number of edits (mutations)	7 (25% quantile), 9 (median), 11 (75% quantile)
$SEQ_{indels,514}$	number of sequences	514
	sequence length (avg.)	34.94
	number of patients	304
	number of edits (mutations,indels)	7 (25% quantile), 10 (median), 13 (75% quantile)

3 Pairwise distance matrix for Cβ atoms in 2b4c

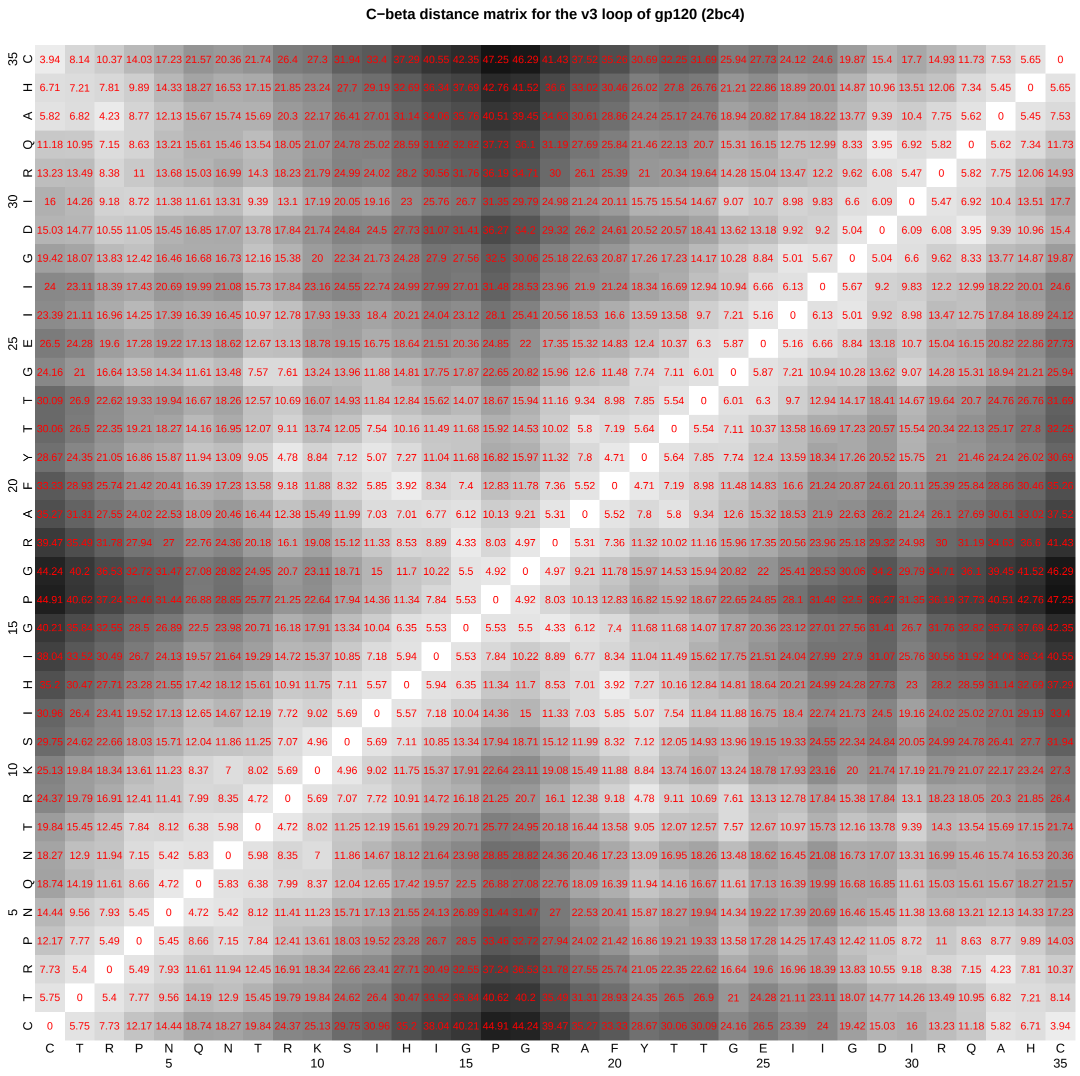

Figure 3: Pairwise distance matrix for Cβ atoms in 2b4c, showing distance relations in the V3 loop.

4 ROC plots

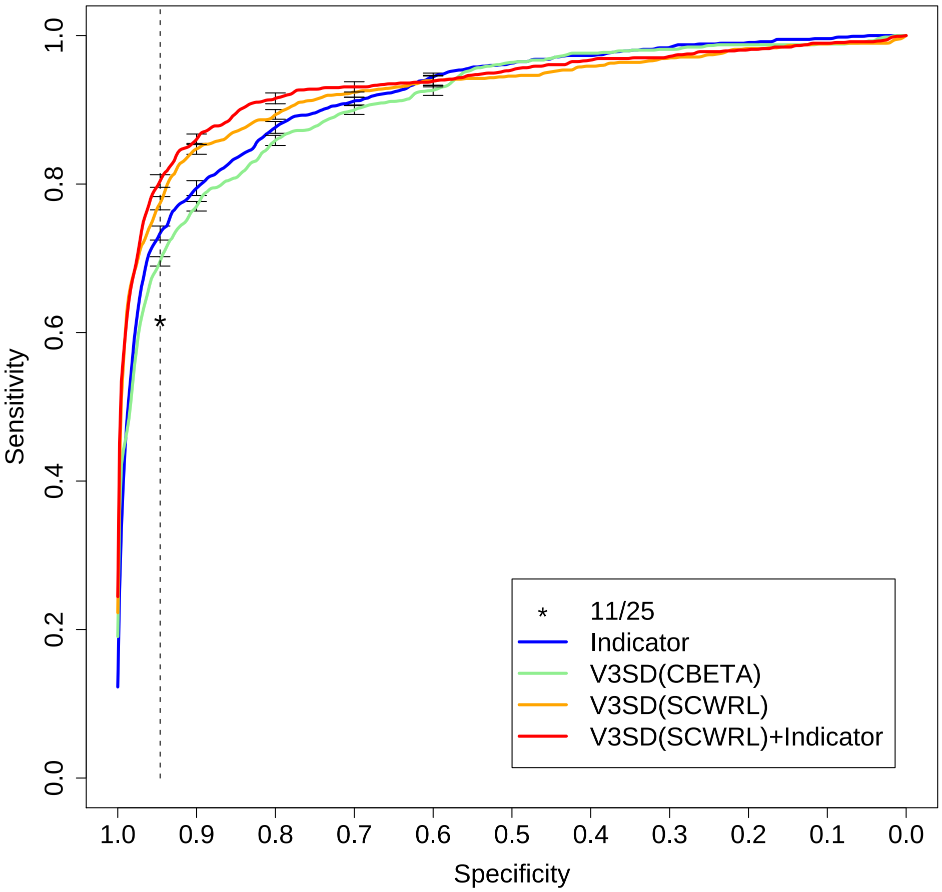

Figure 4: ROC comparison of predictive performance of sequence-based predictions (ChargeRule and Indicator) and structural descriptors ($\mathsf{V3SD}_{\mathrm{cbeta}}$ and $\mathsf{V3SD}_{\mathrm{scwrl}}$) on the data set $\mathrm{SEQ}_{\mathrm{noindels,432}}$. ROC plot is shown over the full range of sensitivity and specificity.

5 Parameter optimization

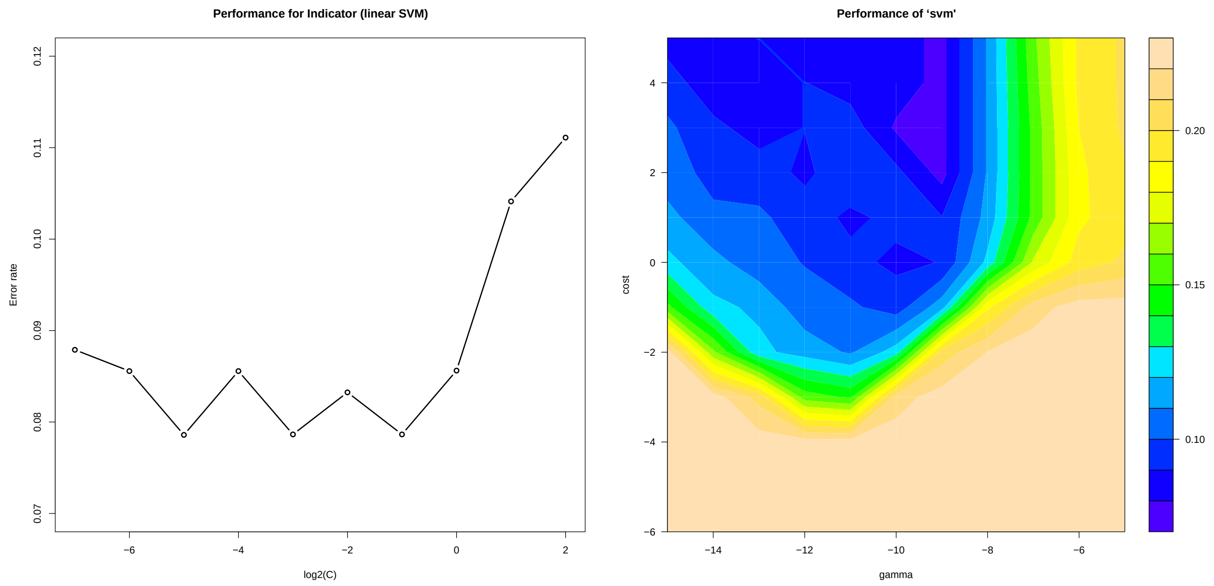

Figure 5: Parameter optimization for linear (for Indicator) and RBF kernels (for V3SD). Performance is averaged over 10 bootstrap samples.

6 Performance for viral variants with indels relative to 2b4c

	11/25 charge rule	Indicator	V3SD(SCWRL)	V3SD(SCWRL)+Indicator
sensitivity at 11/25 spec.	0.5782	0.7182	0.7712	0.8052
AUC	0.7823	0.9004	0.9263	0.9402
Accuracy for cutoff=0.5	0.8482	0.8799	0.8924	0.9053
PPV at 11/25 spec.	0.8174	0.8399	0.8492	0.8543

Table 2: Performance of sequence-based predictions and structural descriptors for the full dataset including sequences with indels relative to 2b4c.

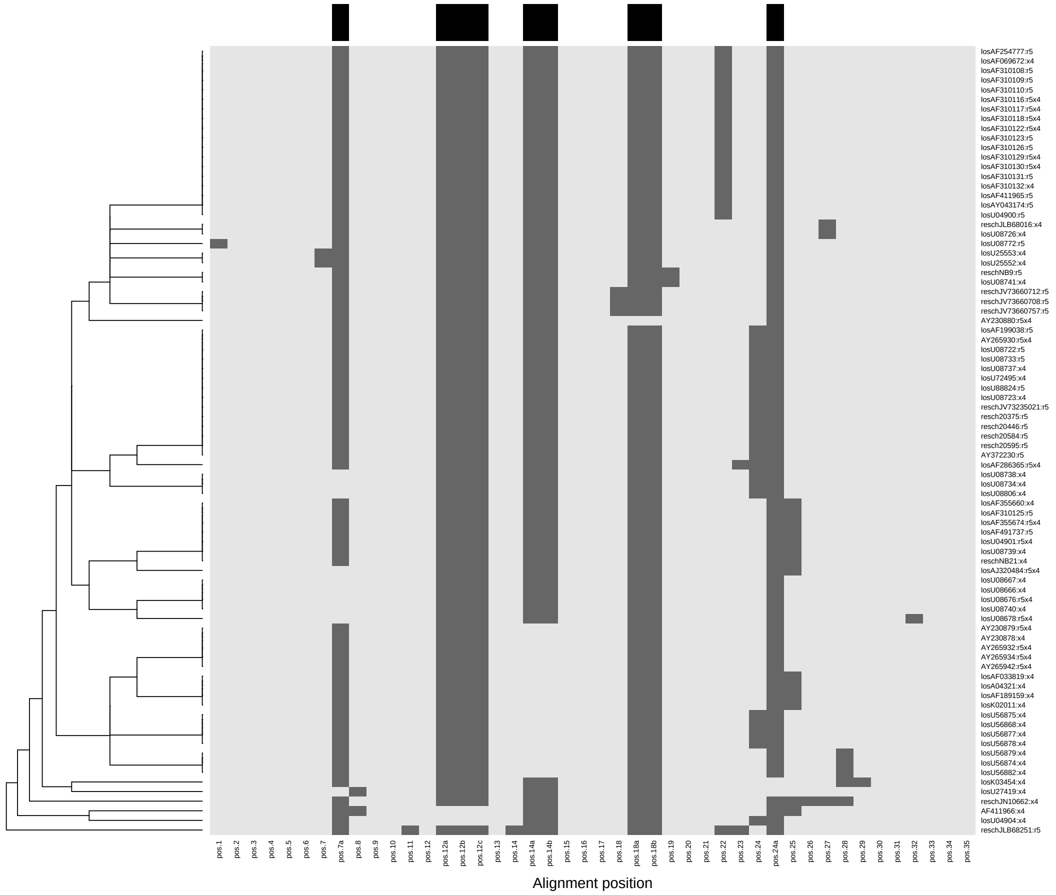

Figure 6: Gap locations; on the x-axis are sequence positions in a multiple alignment, on the y-axis are sequences ordered by patterns of similar insertions and deletions. Dark gray indicates gaps in the alignment, light gray indicates residues. In the first row residues(white) and gaps(black) in the reference V3 loop of 2b4c are marked.

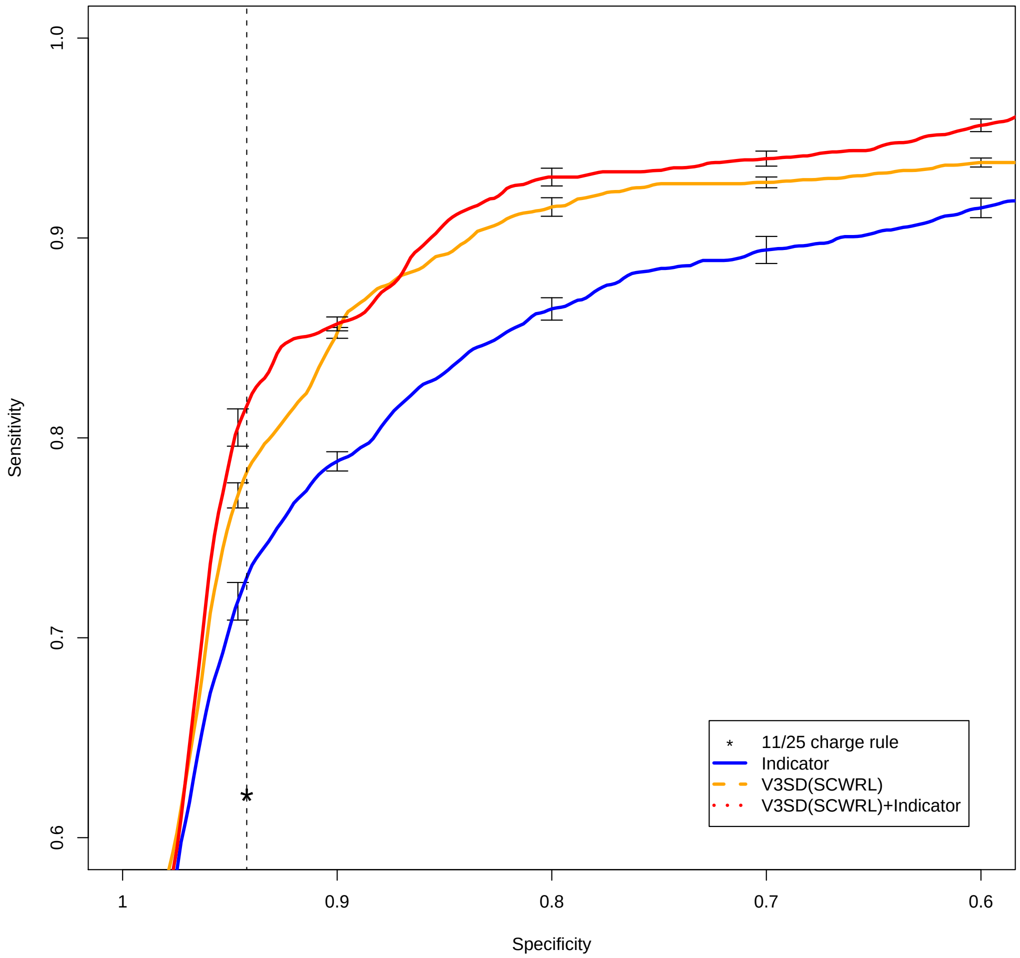

Figure 7: ROC comparison for full dataset including sequences with indels relative to 2b4c.

7 Top-50 descriptor features

	feature	MeanDecreaseGini	wilcoxon.importance	mean.diff	mean.absdiff	variation.ratio
acceptor acceptor 5.5	511	0.0553	49.0000	−1.9388	1.9388	0.2485
acceptor acceptor 6	512	0.0613	49.0000	−2.0417	2.0417	0.2601
acceptor acceptor 6.5	513	0.0450	49.0000	−2.0668	2.0668	0.2626
acceptor acceptor 7	514	0.1988	49.0000	−1.9785	1.9785	0.2454
acceptor acceptor 7.5	515	0.0714	49.0000	−1.7789	1.7789	0.2050
acceptor acceptor 15	530	0.1488	49.0000	−3.3317	3.3317	0.2695
acceptor acceptor 15.5	531	0.0587	49.0000	−3.5702	3.5702	0.2854
acceptor acceptor 16	532	0.1415	49.0000	−3.6593	3.6593	0.2854
acceptor acceptor 16.5	533	0.0935	49.0000	−3.6056	3.6056	0.2644
acceptor acceptor 24	548	0.2045	49.0000	−1.6602	1.6602	0.2138
acceptor acceptor 24.5	549	0.0913	49.0000	−1.4859	1.4859	0.2145
acceptor acceptor 33.5	567	0.1194	49.0000	−0.7166	0.7166	0.2198
acceptor acceptor 34	568	0.0838	49.0000	−0.6136	0.6136	0.1987
donor aliphatic 15.5	331	2.0169	30.3601	6.9703	6.9703	0.4181
donor aliphatic 16	332	1.3078	30.2104	6.3735	6.3735	0.4137
donor aliphatic 15	330	1.7632	29.9028	7.7627	7.7627	0.4217
donor aliphatic 16.5	333	1.3239	28.9347	5.9802	5.9802	0.4086
donor aliphatic 14.5	329	2.3492	28.6020	8.5317	8.5317	0.4212
aliphatic aliphatic 11.5	1223	0.4971	27.7594	1.7459	1.7459	0.4105
aliphatic aliphatic 12	1224	0.6620	27.4380	1.8021	1.8021	0.4101
aliphatic aliphatic 14	1228	0.6408	27.3758	1.7985	1.7985	0.4135
aliphatic aliphatic 11	1222	0.4344	27.3491	1.6332	1.6332	0.3990
donor aliphatic 17	334	1.2743	27.3084	5.6571	5.6571	0.3984
donor aliphatic 3	306	1.5585	27.1577	2.0573	2.0573	0.3992
aliphatic aliphatic 15	1230	0.6855	26.9861	1.4318	1.4318	0.3979
aliphatic aliphatic 14.5	1229	0.8110	26.7527	1.6653	1.6653	0.4170
aliphatic aliphatic 20	1240	1.4043	26.7308	1.3828	1.3828	0.3860
donor aliphatic 3.5	307	0.4127	26.6994	2.5857	2.5857	0.3970
aliphatic aliphatic 20.5	1241	1.1373	26.5423	1.3872	1.3872	0.3890
donor aliphatic 14	328	2.0338	26.3103	8.9657	8.9657	0.4167
donor aliphatic 2.5	305	1.2484	26.2320	1.4180	1.4180	0.3851
aliphatic aliphatic 10.5	1221	0.2924	26.0199	1.5171	1.5171	0.3793
aliphatic aliphatic 12.5	1225	0.2520	25.8772	1.8092	1.8092	0.3996
aliphatic aliphatic 19.5	1239	0.9872	25.8470	1.3268	1.3268	0.3764
aliphatic aliphatic 13.5	1227	0.4309	25.6359	1.8252	1.8252	0.4002
donor aliphatic 17.5	335	0.9559	25.5409	5.3108	5.3108	0.3802
donor aliphatic 4	308	0.5010	25.3739	2.8903	2.8903	0.3856
aliphatic aliphatic 21	1242	0.5683	25.2122	1.2881	1.2881	0.3799
donor aliphatic 26	352	1.6136	25.0075	4.5903	4.5903	0.4096
donor aliphatic 13.5	327	3.0843	24.9482	8.8777	8.8777	0.4101
donor aliphatic 9.5	319	1.2294	24.9015	6.6163	6.6163	0.3988
donor aliphatic 10	320	1.8474	24.7958	6.4527	6.4527	0.3889
donor aliphatic 25.5	351	1.2293	24.7916	4.3823	4.3823	0.4035
aliphatic aliphatic 19	1238	1.2272	24.7498	1.2694	1.2694	0.3644
donor aliphatic 9	318	0.3742	24.7367	6.3566	6.3566	0.3991
donor aliphatic 13	326	0.8888	24.7367	8.2948	8.2948	0.4010
aliphatic aliphatic 15.5	1231	0.2919	24.6823	1.1901	1.1901	0.3514
aliphatic aliphatic 13	1226	0.0552	24.5391	1.8119	1.8119	0.3929
donor aliphatic 12.5	325	1.7345	24.4421	7.4279	7.4279	0.3852
donor aliphatic 18	336	0.5485	24.4128	4.9852	4.9852	0.3569

Table 3: Top-50 descriptor features regarding the Wilcoxon test statistic.

	feature	MeanDecreaseGini	wilcoxon.importance	mean.diff	mean.absdiff	variation.ratio
donor aliphatic 15	330	1.7632	29.9028	7.7627	7.7627	0.4217
donor aliphatic 14.5	329	2.3492	28.6020	8.5317	8.5317	0.4212
donor aliphatic 15.5	331	2.0169	30.3601	6.9703	6.9703	0.4181
aliphatic aliphatic 14.5	1229	0.8110	26.7527	1.6653	1.6653	0.4170
donor aliphatic 14	328	2.0338	26.3103	8.9657	8.9657	0.4167
donor aliphatic 16	332	1.3078	30.2104	6.3735	6.3735	0.4137
aliphatic aliphatic 14	1228	0.6408	27.3758	1.7985	1.7985	0.4135
aliphatic aliphatic 11.5	1223	0.4971	27.7594	1.7459	1.7459	0.4105
donor aliphatic 13.5	327	3.0843	24.9482	8.8777	8.8777	0.4101
aliphatic aliphatic 12	1224	0.6620	27.4380	1.8021	1.8021	0.4101
donor aliphatic 26	352	1.6136	25.0075	4.5903	4.5903	0.4096
donor aliphatic 16.5	333	1.3239	28.9347	5.9802	5.9802	0.4086
donor aliphatic 26.5	353	1.4598	23.9497	4.6795	4.6795	0.4079
donor aliphatic 25.5	351	1.2293	24.7916	4.3823	4.3823	0.4035
donor aliphatic 13	326	0.8888	24.7367	8.2948	8.2948	0.4010
aliphatic aliphatic 13.5	1227	0.4309	25.6359	1.8252	1.8252	0.4002
donor aliphatic 27	354	1.6106	23.0779	4.5924	4.5924	0.3997
aliphatic aliphatic 12.5	1225	0.2520	25.8772	1.8092	1.8092	0.3996
donor aliphatic 3	306	1.5585	27.1577	2.0573	2.0573	0.3992
donor aliphatic 9	318	0.3742	24.7367	6.3566	6.3566	0.3991
aliphatic aliphatic 11	1222	0.4344	27.3491	1.6332	1.6332	0.3990
donor aliphatic 9.5	319	1.2294	24.9015	6.6163	6.6163	0.3988
donor aliphatic 17	334	1.2743	27.3084	5.6571	5.6571	0.3984
aliphatic aliphatic 15	1230	0.6855	26.9861	1.4318	1.4318	0.3979
donor aliphatic 3.5	307	0.4127	26.6994	2.5857	2.5857	0.3970
aliphatic aliphatic 13	1226	0.0552	24.5391	1.8119	1.8119	0.3929
donor aliphatic 25	350	1.1828	24.1744	4.1684	4.1684	0.3910
donor aliphatic 8.5	317	0.1777	23.8916	5.6664	5.6664	0.3893
aliphatic aliphatic 20.5	1241	1.1373	26.5423	1.3872	1.3872	0.3890
donor aliphatic 10	320	1.8474	24.7958	6.4527	6.4527	0.3889
donor aliphatic 27.5	355	1.1899	22.2145	4.3528	4.3528	0.3872
aliphatic aliphatic 20	1240	1.4043	26.7308	1.3828	1.3828	0.3860
donor aliphatic 4	308	0.5010	25.3739	2.8903	2.8903	0.3856
donor aliphatic 12.5	325	1.7345	24.4421	7.4279	7.4279	0.3852
donor aliphatic 2.5	305	1.2484	26.2320	1.4180	1.4180	0.3851
aliphatic aliphatic 25.5	1251	0.3550	19.6725	1.2986	1.2986	0.3833
donor aliphatic 24.5	349	1.4246	23.2493	4.0580	4.0580	0.3802
donor aliphatic 17.5	335	0.9559	25.5409	5.3108	5.3108	0.3802
aliphatic aliphatic 21	1242	0.5683	25.2122	1.2881	1.2881	0.3799
aliphatic aliphatic 10.5	1221	0.2924	26.0199	1.5171	1.5171	0.3793
aliphatic aliphatic 25	1250	0.6757	20.1486	1.2129	1.2129	0.3793
donor aliphatic 24	348	0.9397	22.6118	4.0963	4.0963	0.3772
donor aliphatic 28	356	0.9260	22.0749	4.0333	4.0333	0.3767
aliphatic aliphatic 26	1252	0.1323	19.0358	1.2643	1.2643	0.3766
aliphatic aliphatic 19.5	1239	0.9872	25.8470	1.3268	1.3268	0.3764
donor aliphatic 23.5	347	0.7236	22.5474	4.2531	4.2531	0.3764
aliphatic aliphatic 7.5	1215	0.1757	23.8630	1.9224	1.9224	0.3755
aliphatic aliphatic 8	1216	0.2322	23.4463	1.9401	1.9401	0.3736
aliphatic aliphatic 24.5	1249	0.6991	21.7260	1.0953	1.0953	0.3721
donor aliphatic 28.5	357	1.3069	22.7531	3.7054	3.7054	0.3717

Table 4: Top-50 descriptor features regarding the variation ratio.

	feature	MeanDecreaseGini	wilcoxon.importance	mean.diff	mean.absdiff	variation.ratio
donor aliphatic 13.5	327	3.0843	24.9482	8.8777	8.8777	0.4101
donor aliphatic 14.5	329	2.3492	28.6020	8.5317	8.5317	0.4212
donor aliphatic 14	328	2.0338	26.3103	8.9657	8.9657	0.4167
donor aliphatic 15.5	331	2.0169	30.3601	6.9703	6.9703	0.4181
donor aliphatic 10	320	1.8474	24.7958	6.4527	6.4527	0.3889
donor donor 27	54	1.7704	21.5903	5.4705	5.4705	0.3691
donor aliphatic 15	330	1.7632	29.9028	7.7627	7.7627	0.4217
donor aliphatic 12.5	325	1.7345	24.4421	7.4279	7.4279	0.3852
donor aliphatic 26	352	1.6136	25.0075	4.5903	4.5903	0.4096
donor aliphatic 27	354	1.6106	23.0779	4.5924	4.5924	0.3997
donor aliphatic 3	306	1.5585	27.1577	2.0573	2.0573	0.3992
donor aliphatic 26.5	353	1.4598	23.9497	4.6795	4.6795	0.4079
donor donor 27.5	55	1.4542	21.3859	5.2399	5.2399	0.3592
donor aliphatic 24.5	349	1.4246	23.2493	4.0580	4.0580	0.3802
aliphatic aliphatic 20	1240	1.4043	26.7308	1.3828	1.3828	0.3860
donor donor 26.5	53	1.3440	21.9146	5.6196	5.6196	0.3701
donor aliphatic 10.5	321	1.3275	23.9871	6.1136	6.1136	0.3715
donor aliphatic 16.5	333	1.3239	28.9347	5.9802	5.9802	0.4086
donor aliphatic 16	332	1.3078	30.2104	6.3735	6.3735	0.4137
donor aliphatic 28.5	357	1.3069	22.7531	3.7054	3.7054	0.3717
donor aliphatic 29	358	1.2775	23.2534	3.4077	3.4077	0.3648
donor aliphatic 17	334	1.2743	27.3084	5.6571	5.6571	0.3984
donor aliphatic 2.5	305	1.2484	26.2320	1.4180	1.4180	0.3851
donor aliphatic 9.5	319	1.2294	24.9015	6.6163	6.6163	0.3988
donor aliphatic 25.5	351	1.2293	24.7916	4.3823	4.3823	0.4035
aliphatic aliphatic 19	1238	1.2272	24.7498	1.2694	1.2694	0.3644
donor aliphatic 27.5	355	1.1899	22.2145	4.3528	4.3528	0.3872
donor aliphatic 25	350	1.1828	24.1744	4.1684	4.1684	0.3910
aliphatic aliphatic 20.5	1241	1.1373	26.5423	1.3872	1.3872	0.3890
aliphatic aliphatic 19.5	1239	0.9872	25.8470	1.3268	1.3268	0.3764
donor aliphatic 30	360	0.9823	23.0819	2.8519	2.8519	0.3330
donor aliphatic 17.5	335	0.9559	25.5409	5.3108	5.3108	0.3802
donor aliphatic 24	348	0.9397	22.6118	4.0963	4.0963	0.3772
donor aliphatic 28	356	0.9260	22.0749	4.0333	4.0333	0.3767
aliphatic aliphatic 18.5	1237	0.9055	23.3888	1.2114	1.2114	0.3449
donor aliphatic 13	326	0.8888	24.7367	8.2948	8.2948	0.4010
aliphatic aliphatic 18	1236	0.8548	21.0870	1.1397	1.1397	0.3171
aliphatic aliphatic 14.5	1229	0.8110	26.7527	1.6653	1.6653	0.4170
donor donor 25.5	51	0.7737	22.2334	5.5357	5.5357	0.3515
donor aliphatic 30.5	361	0.7545	22.3987	2.5532	2.5532	0.3095
donor aliphatic 29.5	359	0.7522	23.2820	3.1352	3.1352	0.3514
donor aliphatic 23.5	347	0.7236	22.5474	4.2531	4.2531	0.3764
aliphatic aliphatic 24.5	1249	0.6991	21.7260	1.0953	1.0953	0.3721
aliphatic aliphatic 15	1230	0.6855	26.9861	1.4318	1.4318	0.3979
aliphatic aliphatic 25	1250	0.6757	20.1486	1.2129	1.2129	0.3793
donor donor 26	52	0.6667	22.4097	5.6382	5.6382	0.3636
aliphatic aliphatic 12	1224	0.6620	27.4380	1.8021	1.8021	0.4101
aliphatic aliphatic 24	1248	0.6547	23.7884	1.0075	1.0075	0.3616
aliphatic aliphatic 7	1214	0.6537	23.2414	1.7835	1.7835	0.3681
aliphatic aliphatic 14	1228	0.6408	27.3758	1.7985	1.7985	0.4135

Table 5: Top-50 descriptor features regarding random forest importance (mean decrease in Gini coefficient).

	feature	MeanDecreaseGini	wilcoxon.importance	mean.diff	mean.absdiff	variation.ratio
donor aliphatic 14	328	2.0338	26.3103	8.9657	8.9657	0.4167
donor aliphatic 13.5	327	3.0843	24.9482	8.8777	8.8777	0.4101
donor aliphatic 14.5	329	2.3492	28.6020	8.5317	8.5317	0.4212
donor aliphatic 13	326	0.8888	24.7367	8.2948	8.2948	0.4010
donor aliphatic 15	330	1.7632	29.9028	7.7627	7.7627	0.4217
donor donor 11.5	23	0.1398	18.3921	7.6661	7.6661	0.3029
donor donor 11	22	0.0132	18.6069	7.6312	7.6312	0.3106
donor donor 12	24	0.0172	17.1056	7.5486	7.5486	0.2883
donor donor 13	26	0.0214	15.3132	7.4590	7.4590	0.2749
donor donor 12.5	25	0.0107	16.0999	7.4556	7.4556	0.2777
donor aliphatic 12.5	325	1.7345	24.4421	7.4279	7.4279	0.3852
donor donor 10.5	21	0.0429	18.8855	7.4064	7.4064	0.3074
donor donor 13.5	27	0.0286	14.5563	7.4016	7.4016	0.2743
donor donor 14	28	0.0397	13.9574	7.1069	7.1069	0.2703
donor donor 10	20	0.1787	19.1328	7.0900	7.0900	0.2988
donor aliphatic 15.5	331	2.0169	30.3601	6.9703	6.9703	0.4181
donor donor 9.5	19	0.3206	19.2999	6.7615	6.7615	0.2911
donor donor 14.5	29	0.0291	13.3745	6.6454	6.6454	0.2651
donor aliphatic 9.5	319	1.2294	24.9015	6.6163	6.6163	0.3988
donor aliphatic 12	324	0.1347	23.4090	6.5890	6.5890	0.3641
donor donor 16.5	33	0.0566	14.5048	6.5059	6.5059	0.2861
donor aliphatic 10	320	1.8474	24.7958	6.4527	6.4527	0.3889
donor donor 9	18	0.0631	19.7377	6.4071	6.4071	0.2851
donor donor 17	34	0.0336	13.0376	6.4024	6.4024	0.2688
donor donor 16	32	0.0655	15.2208	6.3824	6.3824	0.2876
donor aliphatic 16	332	1.3078	30.2104	6.3735	6.3735	0.4137
donor aliphatic 9	318	0.3742	24.7367	6.3566	6.3566	0.3991
donor donor 15	30	0.0291	13.7127	6.2922	6.2922	0.2665
donor donor 15.5	31	0.0444	14.4214	6.2297	6.2297	0.2771
donor aliphatic 10.5	321	1.3275	23.9871	6.1136	6.1136	0.3715
donor aliphatic 11.5	323	0.4568	22.5393	6.0436	6.0436	0.3502
donor donor 17.5	35	0.0101	10.7695	6.0326	6.0326	0.2396
donor aliphatic 16.5	333	1.3239	28.9347	5.9802	5.9802	0.4086
donor donor 8.5	17	0.0363	20.0437	5.9789	5.9789	0.2792
donor aliphatic 11	322	0.3016	22.8949	5.9048	5.9048	0.3543
donor aliphatic 8.5	317	0.1777	23.8916	5.6664	5.6664	0.3893
donor aliphatic 17	334	1.2743	27.3084	5.6571	5.6571	0.3984
donor donor 26	52	0.6667	22.4097	5.6382	5.6382	0.3636
donor donor 26.5	53	1.3440	21.9146	5.6196	5.6196	0.3701
donor donor 25.5	51	0.7737	22.2334	5.5357	5.5357	0.3515
donor donor 18	36	0.0180	9.3100	5.5079	5.5079	0.2068
donor donor 27	54	1.7704	21.5903	5.4705	5.4705	0.3691
donor donor 8	16	0.1249	19.7791	5.4526	5.4526	0.2727
donor donor 25	50	0.0477	21.3781	5.3904	5.3904	0.3357
donor aliphatic 17.5	335	0.9559	25.5409	5.3108	5.3108	0.3802
donor donor 24.5	49	0.1361	21.3037	5.2835	5.2835	0.3174
donor donor 27.5	55	1.4542	21.3859	5.2399	5.2399	0.3592
donor donor 24	48	0.1437	21.1007	5.2231	5.2231	0.2988
donor donor 23.5	47	0.1440	20.9491	5.1609	5.1609	0.2813
donor donor 23	46	0.0798	19.2330	5.0659	5.0659	0.2631

Table 6: Top-50 descriptor features regarding the difference in feature means between the coreceptor classes. This measure was not used in the manuscript.

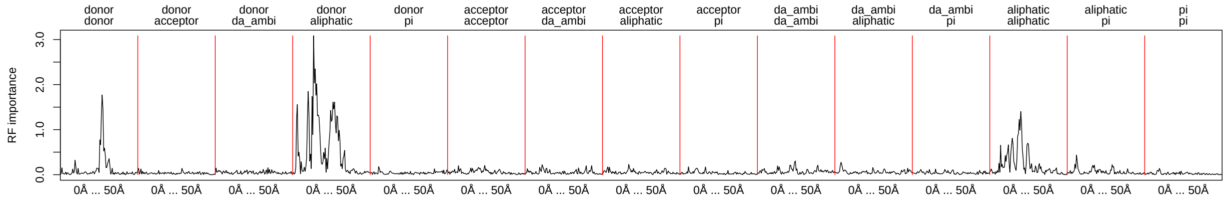

Figure 8: Random forest feature importance measured by the mean decrease in Gini coefficient (repeated from the paper for comparison with Figures 9 and 10).

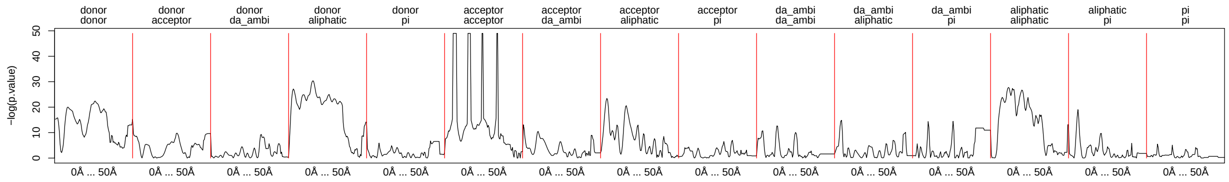

Figure 9: Feature importance measured by the Wilcoxon test statistic.

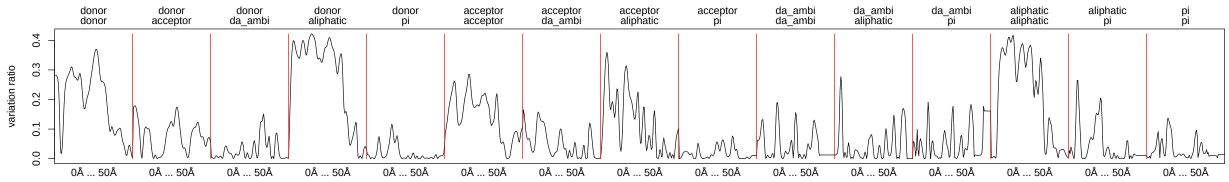

Figure 10: Feature importance measured by the variation ratio.

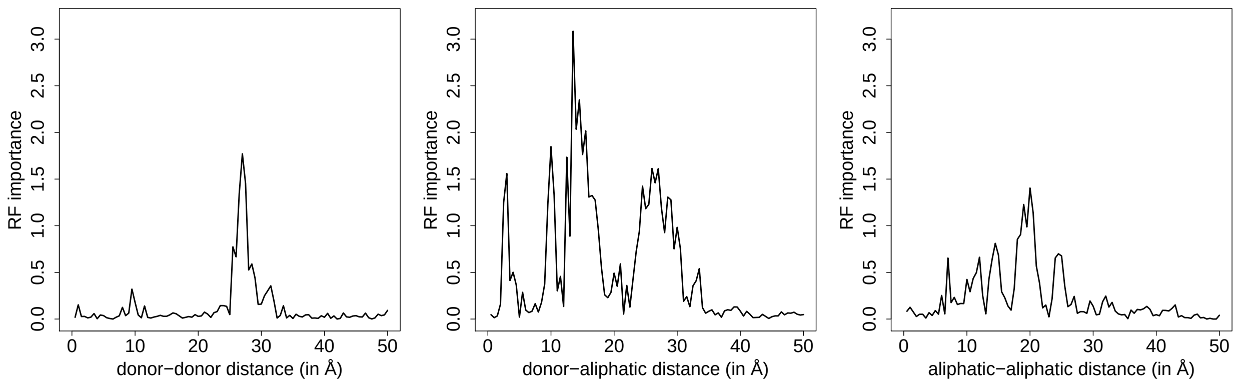

Figure 11: Random forest feature importance measured by the mean decrease in Gini coefficient (important atom types from Figure 8).

8 Profiles of amino acid frequencies and physico-chemical properties

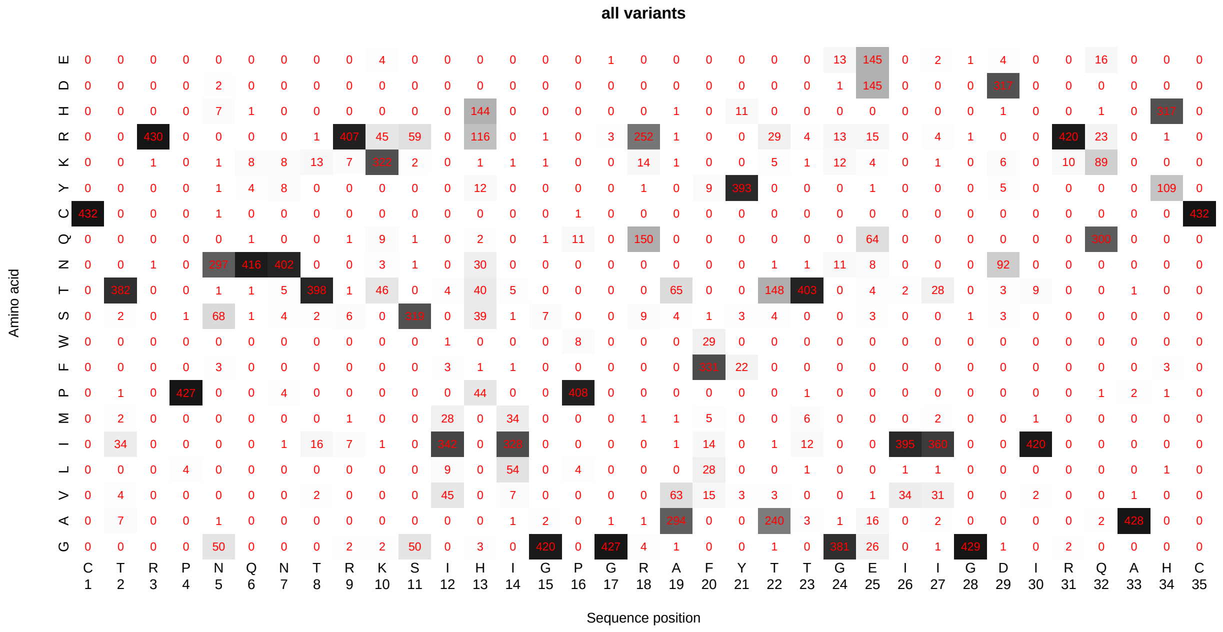

Figure 12: Amino acid frequency profile. Numbers in the cells are absolute frequencies in the data set $SEQ_{noindels,432}$. Gray level indicates relative frequency per position (light gray are rarely observed, dark gray are more common).

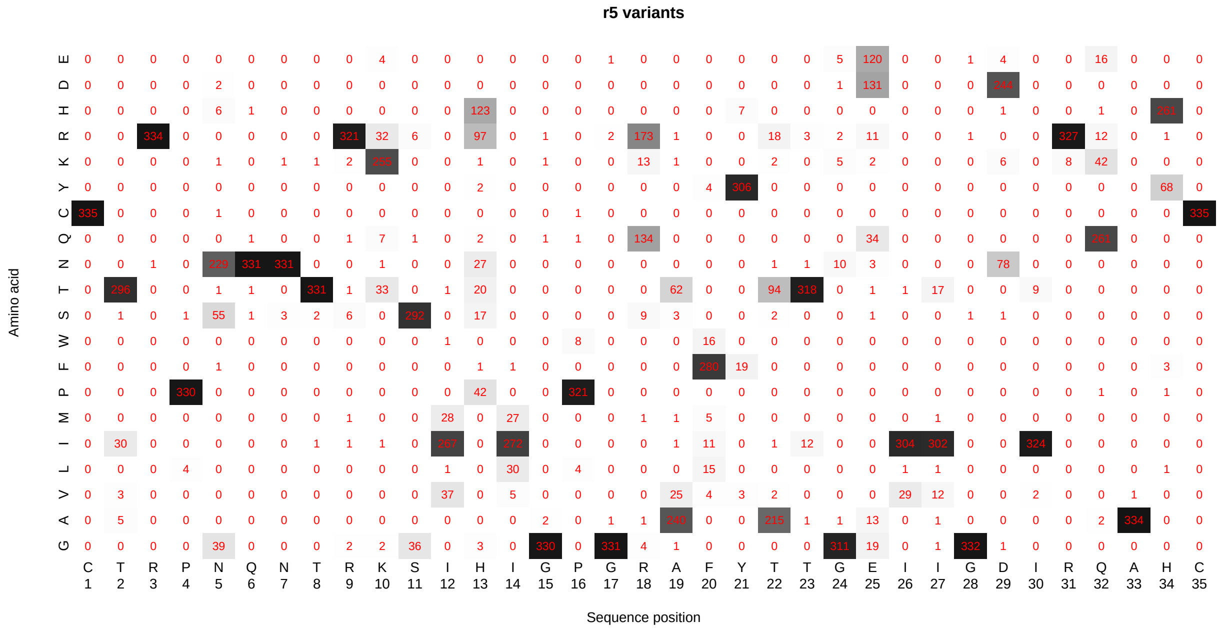

Figure 13: Amino acid frequency profile for R5 variants. Numbers in the cells are absolute frequencies in the data set $SEQ_{\mathrm{noindels},432}$. Gray level indicates relative frequency per position (light gray are rarely observed, dark gray are more common).

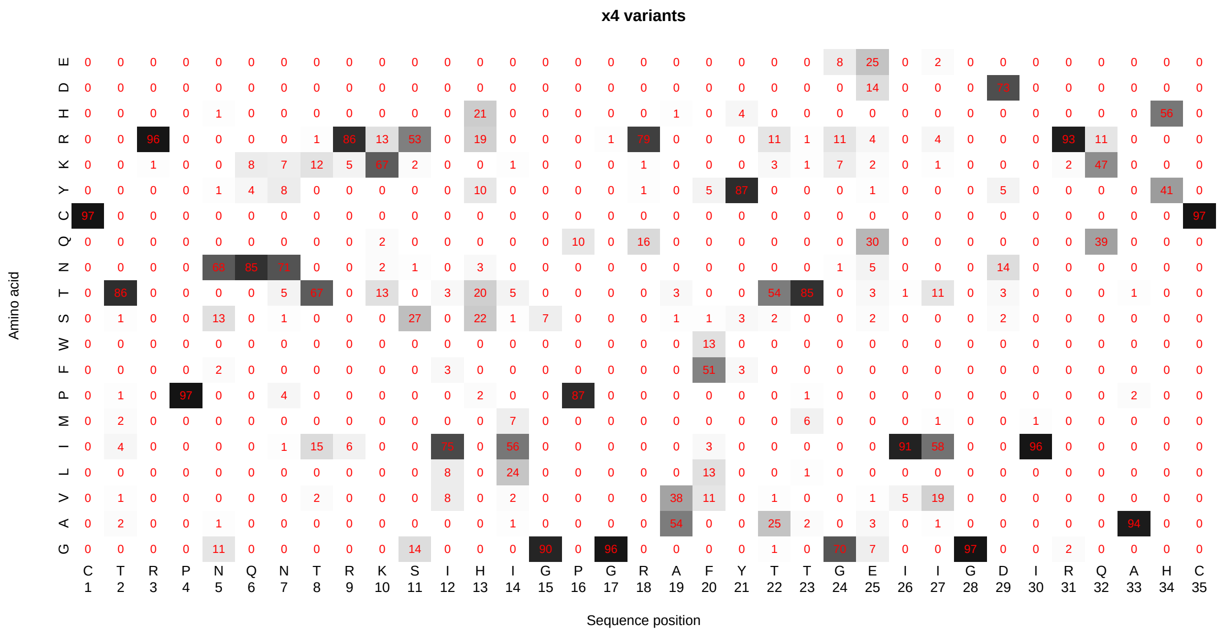

Figure 14: Amino acid frequency profile for X4 variants. Numbers in the cells are absolute frequencies in the data set $SEQ_{noindels,432}$. Gray level indicates relative frequency per position (light gray are rarely observed, dark gray are more common).

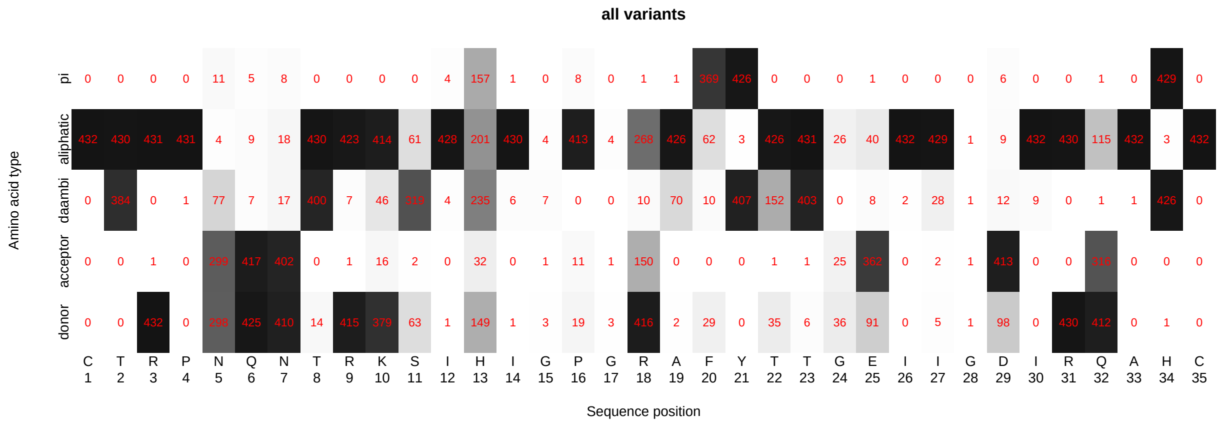

Figure 15: Physico-chemical type frequency profile. Numbers in the cells are absolute frequencies in the data set $SEQ_{noindels,432}$. Gray level indicates relative frequency per position (light gray are rarely observed, dark gray are more common).

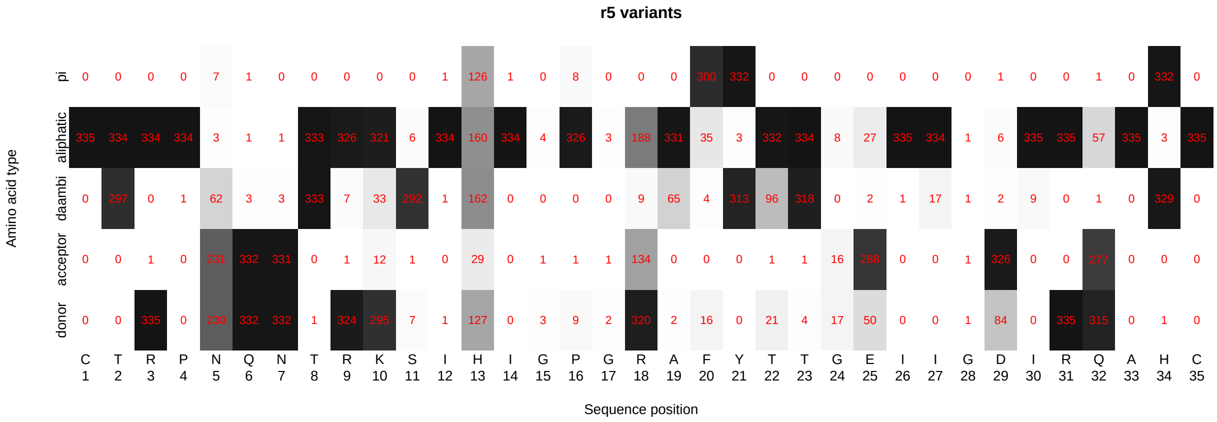

Figure 16: Physico-chemical type frequency profile for R5 variants. Numbers in the cells are absolute frequencies in the data set $\mathrm{SEQ}_{\mathrm{noindels},432}$. Gray level indicates relative frequency per position (light gray are rarely observed, dark gray are more common).

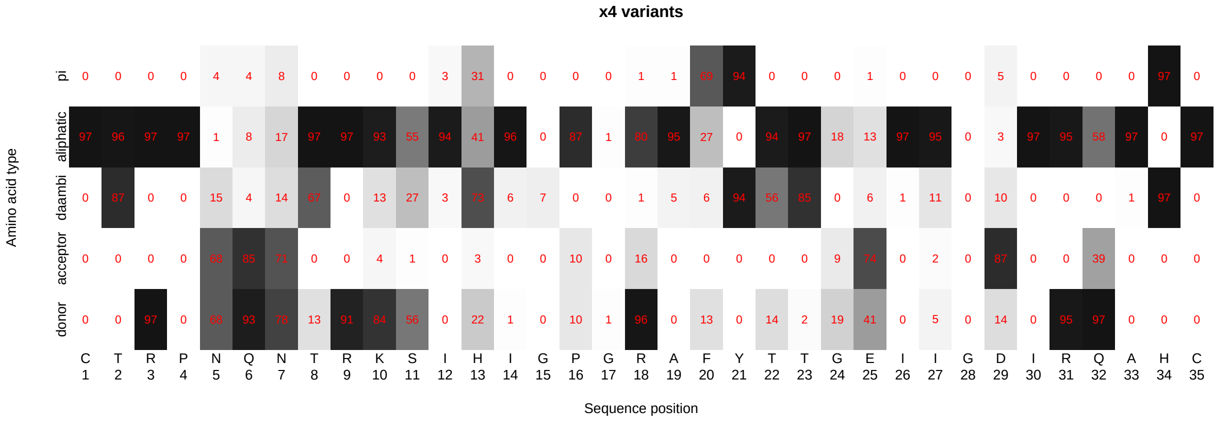

Figure 17: Physico-chemical type frequency profile for X4 variants. Numbers in the cells are absolute frequencies in the data set $\mathrm{SEQ}_{\mathrm{noindels},432}$. Gray level indicates relative frequency per position (light gray are rarely observed, dark gray are more common).

9 Residue-based feature analysis

	X4	rel. X4	R5	rel. R5	rel. X4 - rel. R5
328:328	80.00	0.82	78.00	0.23	0.59
306:306	55.00	0.57	6.00	0.02	0.55
307:306	53.00	0.55	6.00	0.02	0.53
329:328	58.00	0.60	55.00	0.16	0.43
315:315	85.00	0.88	191.00	0.57	0.31
321:321	29.00	0.30	9.00	0.03	0.27
304:303	27.00	0.28	3.00	0.01	0.27
324:323	35.00	0.36	31.00	0.09	0.27
322:321	18.00	0.19	8.00	0.02	0.16
323:323	21.00	0.22	19.00	0.06	0.16
303:303	81.00	0.84	332.00	0.99	−0.16
317:316	41.00	0.42	91.00	0.27	0.15
319:316	23.00	0.24	38.00	0.11	0.12
317:315	13.00	0.13	8.00	0.02	0.11
308:317	14.00	0.14	15.00	0.04	0.10
309:308	4.00	0.04	45.00	0.13	−0.09
320:319	15.00	0.15	23.00	0.07	0.09
312:309	87.00	0.90	329.00	0.98	−0.09
319:319	14.00	0.14	20.00	0.06	0.08
307:307	0.00	0.00	28.00	0.08	−0.08
301:301	8.00	0.08	0.00	0.00	0.08
318:317	16.00	0.16	31.00	0.09	0.07
302:302	7.00	0.07	1.00	0.00	0.07
321:303	8.00	0.08	5.00	0.01	0.07
314:313	87.00	0.90	322.00	0.96	−0.06
309:309	6.00	0.06	0.00	0.00	0.06
317:308	9.00	0.09	51.00	0.15	−0.06
303:302	5.00	0.05	0.00	0.00	0.05
308:308	39.00	0.40	119.00	0.36	0.05
300:302	4.00	0.04	0.00	0.00	0.04

Table 7: donor-aliphatic from 3.5 to 4.5

	X4	rel. X4	R5	rel. R5	rel. X4 - rel. R5
315:306	213.00	2.20	24.00	0.07	2.12
306:315	236.00	2.43	207.00	0.62	1.82
306:303	259.00	2.67	352.00	1.05	1.62
306:316	257.00	2.65	350.00	1.04	1.60
306:320	257.00	2.65	353.00	1.05	1.60
306:309	255.00	2.63	353.00	1.05	1.58
306:319	250.00	2.58	347.00	1.04	1.54
304:306	154.00	1.59	16.00	0.05	1.54
306:313	132.00	1.36	19.00	0.06	1.30
327:328	164.00	1.69	168.00	0.50	1.19
306:304	113.00	1.16	12.00	0.04	1.13
306:307	108.00	1.11	14.00	0.04	1.07
301:306	106.00	1.09	12.00	0.04	1.06
306:305	98.00	1.01	10.00	0.03	0.98
302:306	96.00	0.99	12.00	0.04	0.95
300:306	92.00	0.95	11.00	0.03	0.92
321:306	87.00	0.90	7.00	0.02	0.88
298:328	116.00	1.20	109.00	0.33	0.87
301:328	114.00	1.18	113.00	0.34	0.84
319:306	82.00	0.85	6.00	0.02	0.83
300:328	99.00	1.02	81.00	0.24	0.78
302:328	103.00	1.06	113.00	0.34	0.72
303:306	70.00	0.72	7.00	0.02	0.70
315:304	346.00	3.57	969.00	2.89	0.67
327:321	72.00	0.74	24.00	0.07	0.67
298:302	66.00	0.68	4.00	0.01	0.67
317:306	65.00	0.67	6.00	0.02	0.65
298:321	72.00	0.74	31.00	0.09	0.65
315:320	351.00	3.62	999.00	2.98	0.64
315:309	252.00	2.60	666.00	1.99	0.61

Table 8: donor-aliphatic from 9 to 18

	X4	rel. X4	R5	rel. R5	rel. X4 - rel. R5
298:306	220.00	2.27	24.00	0.07	2.20
327:306	210.00	2.16	22.00	0.07	2.10
306:328	180.00	1.86	57.00	0.17	1.69
306:297	249.00	2.57	315.00	0.94	1.63
306:298	161.00	1.66	19.00	0.06	1.60
306:329	257.00	2.65	354.00	1.06	1.59
306:327	156.00	1.61	19.00	0.06	1.55
306:323	237.00	2.44	337.00	1.01	1.44
328:306	132.00	1.36	12.00	0.04	1.33
327:315	236.00	2.43	563.00	1.68	0.75
298:317	106.00	1.09	140.00	0.42	0.67
325:306	66.00	0.68	6.00	0.02	0.66
327:317	102.00	1.05	140.00	0.42	0.63
315:326	351.00	3.62	1001.00	2.99	0.63
315:302	62.00	0.64	4.00	0.01	0.63
315:323	343.00	3.54	997.00	2.98	0.56
297:306	55.00	0.57	6.00	0.02	0.55
329:306	55.00	0.57	6.00	0.02	0.55
330:306	55.00	0.57	6.00	0.02	0.55
315:299	244.00	2.52	661.00	1.97	0.54
306:326	54.00	0.56	6.00	0.02	0.54
301:315	157.00	1.62	374.00	1.12	0.50
328:317	68.00	0.70	74.00	0.22	0.48
308:328	73.00	0.75	102.00	0.30	0.45
318:328	58.00	0.60	54.00	0.16	0.44
300:315	138.00	1.42	331.00	0.99	0.43
307:328	58.00	0.60	57.00	0.17	0.43
317:328	58.00	0.60	57.00	0.17	0.43
308:323	149.00	1.54	655.00	1.96	−0.42
298:308	142.00	1.46	618.00	1.84	−0.38

Table 9: donor-aliphatic from 23 to 30

	X4	rel. X4	R5	rel. R5	rel. X4 - rel. R5
315:328	137.00	1.41	92.00	0.27	1.14
306:296	53.00	0.55	6.00	0.02	0.53
306:331	53.00	0.55	7.00	0.02	0.53
312:328	58.00	0.60	54.00	0.16	0.44
315:298	79.00	0.81	173.00	0.52	0.30
298:315	80.00	0.82	188.00	0.56	0.26
330:316	3.00	0.03	65.00	0.19	−0.16
328:315	69.00	0.71	184.00	0.55	0.16
297:316	92.00	0.95	266.00	0.79	0.15
331:317	11.00	0.11	4.00	0.01	0.10
319:297	14.00	0.14	20.00	0.06	0.08
325:309	11.00	0.11	60.00	0.18	−0.07
326:313	87.00	0.90	321.00	0.96	−0.06
298:309	5.00	0.05	0.00	0.00	0.05
329:309	84.00	0.87	307.00	0.92	−0.05
308:329	3.00	0.03	27.00	0.08	−0.05
308:297	3.00	0.03	25.00	0.07	−0.04
316:297	7.00	0.07	38.00	0.11	−0.04
323:309	4.00	0.04	0.00	0.00	0.04
315:327	5.00	0.05	4.00	0.01	0.04
300:313	60.00	0.62	220.00	0.66	−0.04
328:309	7.00	0.07	12.00	0.04	0.04
315:325	0.00	0.00	9.00	0.03	−0.03
296:319	3.00	0.03	2.00	0.01	0.02
314:327	95.00	0.98	335.00	1.00	−0.02
312:325	0.00	0.00	6.00	0.02	−0.02
322:331	8.00	0.08	22.00	0.07	0.02
314:299	97.00	1.00	330.00	0.99	0.01
309:297	87.00	0.90	296.00	0.88	0.01
324:313	0.00	0.00	4.00	0.01	−0.01

Table 10: donor-aliphatic from 32.5 to 33.5

	X4	rel. X4	R5	rel. R5	rel. X4 - rel. R5
306:307	53.00	0.55	6.00	0.02	0.53
305:306	42.00	0.43	4.00	0.01	0.42
319:320	54.00	0.56	94.00	0.28	0.28
307:309	54.00	0.56	252.00	0.75	−0.20
308:315	20.00	0.21	6.00	0.02	0.19
321:322A	18.00	0.19	8.00	0.02	0.16
316:319	3.00	0.03	59.00	0.18	−0.15
307:316	54.00	0.56	228.00	0.68	−0.12
315:317	14.00	0.14	7.00	0.02	0.12
316:317	13.00	0.13	4.00	0.01	0.12
302:305	11.00	0.11	1.00	0.00	0.11
317:319	11.00	0.11	1.00	0.00	0.11
304:307	11.00	0.11	2.00	0.01	0.11
304:305	30.00	0.31	68.00	0.20	0.11
308:316	2.00	0.02	41.00	0.12	−0.10
322A:323	21.00	0.22	39.00	0.12	0.10
307:317	14.00	0.14	15.00	0.04	0.10
298:328	11.00	0.11	12.00	0.04	0.08
321:326	7.00	0.07	4.00	0.01	0.06
307:319	2.00	0.02	27.00	0.08	−0.06
308:309	19.00	0.20	46.00	0.14	0.06
322:322A	9.00	0.09	13.00	0.04	0.05
299:302	5.00	0.05	0.00	0.00	0.05
302:303	4.00	0.04	0.00	0.00	0.04
301:302	4.00	0.04	0.00	0.00	0.04
307:308	18.00	0.19	74.00	0.22	−0.04
297:298	8.00	0.08	38.00	0.11	−0.03
297:329	8.00	0.08	38.00	0.11	−0.03
329:331	94.00	0.97	335.00	1.00	−0.03
325:326	3.00	0.03	0.00	0.00	0.03

Table 11: aliphatic-aliphatic from 6.5 to 7.5

	X4	rel. X4	R5	rel. R5	rel. X4 - rel. R5
306:316	53.00	0.55	6.00	0.02	0.53
322A:328	58.00	0.60	57.00	0.17	0.43
297:328	56.00	0.58	51.00	0.15	0.43
319:322A	25.00	0.26	215.00	0.64	−0.38
323:327	60.00	0.62	304.00	0.91	−0.29
303:319	27.00	0.28	22.00	0.07	0.21
303:328	21.00	0.22	4.00	0.01	0.20
303:306	20.00	0.21	1.00	0.00	0.20
323:328	22.00	0.23	12.00	0.04	0.19
297:302	17.00	0.18	1.00	0.00	0.17
305:308	21.00	0.22	130.00	0.39	−0.17
299:321	18.00	0.19	7.00	0.02	0.16
298:303	80.00	0.82	331.00	0.99	−0.16
321:327	18.00	0.19	8.00	0.02	0.16
303:327	15.00	0.15	1.00	0.00	0.15
307:321	17.00	0.18	8.00	0.02	0.15
304:308	20.00	0.21	20.00	0.06	0.15
305:321	15.00	0.15	6.00	0.02	0.14
306:319	13.00	0.13	2.00	0.01	0.13
298:302	12.00	0.12	1.00	0.00	0.12
304:316	37.00	0.38	89.00	0.27	0.12
303:308	2.00	0.02	42.00	0.13	−0.10
316:321	11.00	0.11	3.00	0.01	0.10
305:317	15.00	0.15	20.00	0.06	0.09
317:321	9.00	0.09	0.00	0.00	0.09
301:326	8.00	0.08	0.00	0.00	0.08
304:322	10.00	0.10	7.00	0.02	0.08
303:317	18.00	0.19	35.00	0.10	0.08
301:319	7.00	0.07	0.00	0.00	0.07
307:313	67.00	0.69	254.00	0.76	−0.07

Table 12: aliphatic-aliphatic from 12 to 14

	X4	rel. X4	R5	rel. R5	rel. X4 - rel. R5
299:306	55.00	0.57	6.00	0.02	0.55
306:326	52.00	0.54	6.00	0.02	0.52
305:328	48.00	0.49	51.00	0.15	0.34
303:315	80.00	0.82	185.00	0.55	0.27
315:322A	80.00	0.82	187.00	0.56	0.27
305:315	76.00	0.78	183.00	0.55	0.24
296:303	18.00	0.19	1.00	0.00	0.18
302:331	17.00	0.18	1.00	0.00	0.17
316:322A	3.00	0.03	63.00	0.19	−0.16
303:331	15.00	0.15	1.00	0.00	0.15
316:326	92.00	0.95	269.00	0.80	0.15
299:319	80.00	0.82	310.00	0.93	−0.10
302:320	10.00	0.10	1.00	0.00	0.10
308:322A	19.00	0.20	98.00	0.29	−0.10
302:308	9.00	0.09	0.00	0.00	0.09
317:326	14.00	0.14	20.00	0.06	0.08
296:301	8.00	0.08	1.00	0.00	0.08
319:327	89.00	0.92	330.00	0.99	−0.07
305:322	13.00	0.13	25.00	0.07	0.06
297:321	7.00	0.07	5.00	0.01	0.06
309:321	7.00	0.07	5.00	0.01	0.06
302:319	5.00	0.05	0.00	0.00	0.05
298:322	9.00	0.09	14.00	0.04	0.05
303:309	75.00	0.77	276.00	0.82	−0.05
307:322A	3.00	0.03	27.00	0.08	−0.05
301:323	5.00	0.05	1.00	0.00	0.05
309:322	5.00	0.05	3.00	0.01	0.04
304:313	87.00	0.90	312.00	0.93	−0.03
302:323	3.00	0.03	0.00	0.00	0.03
320:325	3.00	0.03	0.00	0.00	0.03

Table 13: aliphatic-aliphatic from 19 to 21

	X4	rel. X4	R5	rel. R5	rel. X4 - rel. R5
306:327	49.00	0.51	5.00	0.01	0.49
306:323	41.00	0.42	5.00	0.01	0.41
319:329	25.00	0.26	215.00	0.64	−0.38
296:323	59.00	0.61	303.00	0.90	−0.30
323:331	59.00	0.61	303.00	0.90	−0.30
315:326	79.00	0.81	187.00	0.56	0.26
303:313	72.00	0.74	318.00	0.95	−0.21
299:316	92.00	0.95	262.00	0.78	0.17
315:323	23.00	0.24	24.00	0.07	0.17
308:323	11.00	0.11	91.00	0.27	−0.16
302:315	10.00	0.10	0.00	0.00	0.10
298:317	11.00	0.11	4.00	0.01	0.10
317:327	11.00	0.11	4.00	0.01	0.10
297:323	74.00	0.76	279.00	0.83	−0.07
309:326	83.00	0.86	306.00	0.91	−0.06
309:322A	5.00	0.05	1.00	0.00	0.05
297:322	3.00	0.03	0.00	0.00	0.03
307:325	3.00	0.03	0.00	0.00	0.03
306:325	2.00	0.02	0.00	0.00	0.02
307:328	3.00	0.03	5.00	0.01	0.02
296:304	92.00	0.95	323.00	0.96	−0.02
319:328	2.00	0.02	2.00	0.01	0.01
305:313	0.00	0.00	4.00	0.01	−0.01
316:327	2.00	0.02	3.00	0.01	0.01
297:306	1.00	0.01	0.00	0.00	0.01
307:323	1.00	0.01	0.00	0.00	0.01
307:329	1.00	0.01	0.00	0.00	0.01
297:318	0.00	0.00	3.00	0.01	−0.01
313:322	3.00	0.03	13.00	0.04	−0.01
296:322A	1.00	0.01	1.00	0.00	0.01

Table 14: aliphatic-aliphatic from 24.5 to 25.5

	X4	rel. X4	R5	rel. R5	rel. X4 - rel. R5
298:306	106.00	1.09	12.00	0.04	1.06
306:328	68.00	0.70	11.00	0.03	0.67
297:306	55.00	0.57	7.00	0.02	0.55
306:329	53.00	0.55	6.00	0.02	0.53
306:330	53.00	0.55	7.00	0.02	0.53
306:327	52.00	0.54	7.00	0.02	0.52
298:308	73.00	0.75	382.00	1.14	−0.39
315:325	175.00	1.80	492.00	1.47	0.34
315:326	272.00	2.80	827.00	2.47	0.34
300:315	261.00	2.69	806.00	2.41	0.28
308:328	38.00	0.39	223.00	0.67	−0.27
297:322	34.00	0.35	45.00	0.13	0.22
308:327	115.00	1.19	450.00	1.34	−0.16
308:325	17.00	0.18	106.00	0.32	−0.14
306:325	11.00	0.11	0.00	0.00	0.11
306:323	8.00	0.08	0.00	0.00	0.08
315:323	8.00	0.08	0.00	0.00	0.08
301:315	10.00	0.10	8.00	0.02	0.08
317:327	12.00	0.12	16.00	0.05	0.08
316:328	108.00	1.11	350.00	1.04	0.07
296:304	86.00	0.89	318.00	0.95	−0.06
298:319	11.00	0.11	18.00	0.05	0.06
319:329	11.00	0.11	18.00	0.05	0.06
307:323	5.00	0.05	0.00	0.00	0.05
308:323	5.00	0.05	0.00	0.00	0.05
319:328	11.00	0.11	21.00	0.06	0.05
322:331	5.00	0.05	3.00	0.01	0.04
316:323	4.00	0.04	0.00	0.00	0.04
305:314	13.00	0.13	32.00	0.10	0.04
309:327	283.00	2.92	989.00	2.95	−0.03

Table 15: donor-donor from 27.5 to 28.5

10 Visualization of residue-based feature analysis

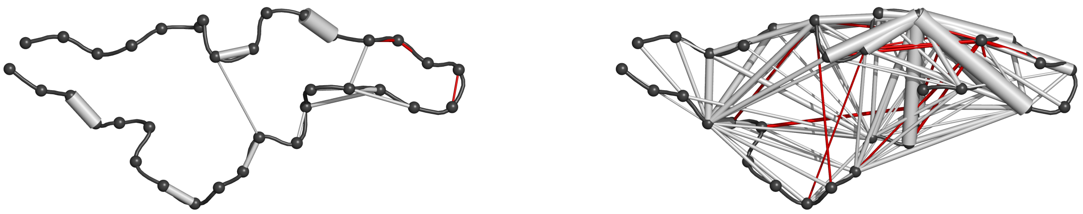

Figure 18: Donor-aliphatic distance ranges 4Å ± 0.5Å and 9Å to 18Å.

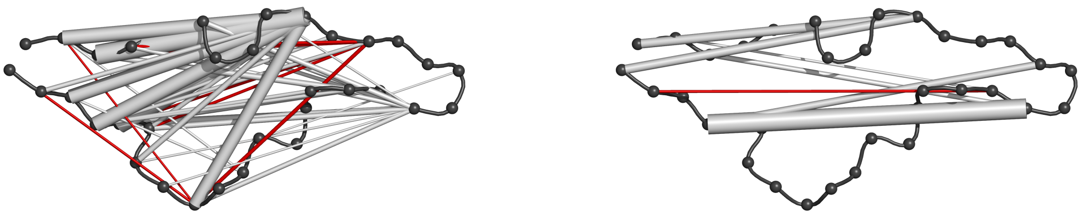

Figure 19: Donor-aliphatic distance ranges 23Å to 30Å and 33Å ± 0.5Å

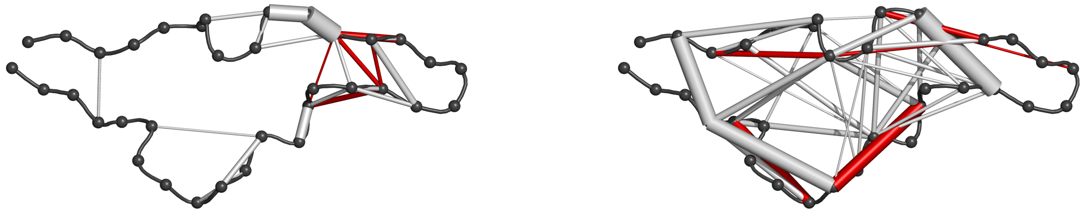

Figure 20: Aliphatic-aliphatic distance ranges 7Å ± 0.5Å and 12Å to 14Å.

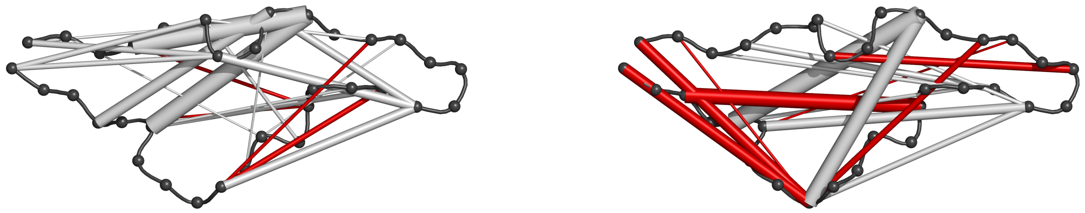

Figure 21: Aliphatic-aliphatic distance ranges 19Å to 21Å and 25Å ± 0.5Å

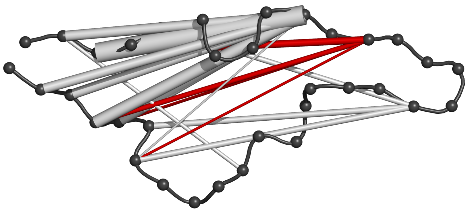

Figure 22: Donor-donor atoms in the distance of 28Å ± 0.5Å.
